# Comparative analysis of the transcriptome of the Amazonian fish species *Colossoma macropomum* (tambaqui) and hybrid tambacu by next generation sequencing

**DOI:** 10.1371/journal.pone.0212755

**Published:** 2019-02-25

**Authors:** Fátima Gomes, Luciana Watanabe, João Vianez, Márcio Nunes, Jedson Cardoso, Clayton Lima, Horacio Schneider, Iracilda Sampaio

**Affiliations:** 1 Institute of Coastal Studies, Laboratory of Genetics and Molecular Biology, Universidade Federal do Pará, Campus de Bragança, Alameda Leandro Ribeiro, Bragança, PA, Brazil; 2 Center for Technological Innovation, Evandro Chagas Institute, Ministry of Health, Ananindeua, PA, Brazil; 3 Postgraduate Program in Virology (PPGV), Evandro Chagas Institute, Ministry of Health, Ananindeua, PA, Brazil; University of Lausanne, SWITZERLAND

## Abstract

**Background:**

The *C*. *macropomum* is a characiform fish from the Amazon basin that has been hybridized with other pacu species to produce commercial hybrids, such as the tambacu. However, little is known of the functional genomics of the parental species or these hybrid forms. The transcriptome of *C*. *macropomum* and tambacu were sequenced using 454 Roche platform (pyrosequencing) techniques to characterize the domains of Gene Ontology (GO) and to evaluate the levels of gene expression in the two organisms.

**Results:**

The 8,188,945 reads were assembled into 400,845 contigs. A total of 58,322 contigs were annotated with a predominance of biological processes for both organisms, as determined by Gene Ontology (GO). Similar numbers of metabolic pathways were identified in both the *C*. *macropomum* and the tambacu, with the metabolism category presenting the largest number of transcripts. The BUSCO analysis indicated that our assembly was more than 40% complete. We identified 21,986 genes for the two fishes. The P and Log2FC values indicated significant differences in the levels of gene expression, with a total of 600 up-regulated genes.

**Conclusion:**

In spite of the lack of a reference genome, the functional annotation was successful, and confirmed a considerable difference in the specificity and levels of gene expression between the two organisms. This report provides a comprehensive baseline for the genetic management of these commercially important fishes, in particular for the identification of specific genes that may represent markers involved in the immunity, growth, and fertility of these organisms, with potential practical applications in aquaculture management.

## Background

Next Generation Sequencing (NGS) technologies have provided a range of potential markers for both model and non-model species through the discovery of new genes from an enormous database [1–3]. Over the past few years, NGS has been used increasingly to characterize the transcripts of a number of different species and tissues, in addition to contributing to the evaluation of gene expression in commercial lineages. Fish are especially important, given that they comprise a highly diversified group, with many economically important species, but little information on their functional genomics. The FLX (GS FLX) Genome sequencing system, which uses the 454 sequencing approach, is one of the most widely-used and successful approaches to the evaluation of the fish transcriptome [4–6].

The tambaqui (*Colossoma macropomum*) is an important fishery resource in the Amazon region, and is distributed naturally in both the Amazon and Orinoco river basins. Given its aquacultural performance, due to its rapid growth and robustness, the *C*. *macropomum* is currently the most common species raised on Brazilian fish farms [7, 8], and is becoming increasingly popular in other tropical countries. As the second largest South American freshwater fish, reaching 90 cm and 30 kg, the *C*. *macropomum* is especially suitable for tropical aquaculture, especially as it is also extremely fertile and robust, and can adapt easily to polyculture systems [7]. This species has also often been hybridized with other pacus, in particular *Piaractus mesopotamicus* [9, 10], producing a hybrid known as the tambacu. The tambacu results from the induced crossing of the female *C*. *macropomum* with the male *P*. *mesopotamicus*, and has been raised ever more intensively in recent years, replacing its parental species in tropical aquaculture operations [8].

The few comparative studies available for the *C*. *macropomum* and tambacu have focused on growth, genetics, biochemistry, and the immune system [11–13], while no comparative analyses of their functional genomics have yet been published. In fact, few data are available on the functional genomics of *C*. *macropomum*. However, Prado-Lima and Val [14] did analyze the transcriptome of the muscle of juvenile t *C*. *macropomum*, subjected to variation in temperature. In this experiment, significant differences were observed in the expression of specific groups of genes in responses to changes in the ambient conditions. Gomes et al. [15] characterized the miRNAs of the liver and muscle of juvenile *C*. *macropomum*, and identified a number of conserved miRNAs that were shared between the two types of tissue, albeit with distinct levels of expression. Studying 30 *C*. *macropomum* specimens, Martinez et al. [16] analyzed nuclear Single Nucleotide Polymorphisms (SNPs) using Next Generation Sequencing-based genotyping. Using the genotyping by sequencing method, Nunes et al. [17] identified a large number of SNPs in *C*. *macropomum*, and constructed a high-resolution genetic linkage map for a full-sibling family of 124 individuals, including their parents. Apart from these studies, there are no data on the transcriptome of the tambacu or any comparative analyses involving its ancestral species.

The transcriptome is the initial product of genome expression and is the principal focus of the investigation of gene function [18, 19]. Thus, we compared the transcriptome of *C*. *macropomum* and tambacu to characterize the Gene Ontology (GO) domains and metabolic pathways, as well as evaluating the levels of gene expression in the two organisms. Two types of tissue–skin and muscle–were chosen for these analyses. The skin represents the main defensive barrier of a vertebrate, and presents unique biological processes related to its complex structure and cell composition [20, 21]. Skeletal muscle represents about half of the body mass of a fish, and is associated with the swimming capabilities of these animals [22]. The results of this study are important for the conservation of the species, and the markers may also have practical applications for management of farmed stocks.

## Material and methods

### Ethics statement

The samples analyzed in the present study were obtained from fish farms and collected with the permission of the Brazilian federal environmental, authorized by the Chico Mendes Institute for Biodiversity (agency ICMBio) through license number 12.773–1 granted to Iracilda Sampaio. The animal experimentation conducted was in accordance with the National control Board of Animal Experimentation (CONCEA), Brazilian federal law no. 11.794, and the International guidelines defined by the U.K. Animals (Scientific Procedures) Act, 1986. The specimens were then euthanized by immersion in anesthetic Benzocaine Hydrochloride, as recommended by the Brazilian Federal Council of Veterinary Medicine (federal law 5,517, resolution CFMV no. 1000/2012). All efforts were made to minimize the intensity and duration of distress and exposure to pain.

### Sample collection

The *C*. *macropomum* and tambacu specimens were collected from the Andrera fish farm in Bonito, in Pará state, Brazil. All specimens were identified prior to collection, based on morphological traits, such as the shape of the body, and the number of rakers on the first gill arch. However, given the considerable morphological similarities between the *C*. *macropomum* and its hybrids, genetic tests were conducted to confirm the identity of the specimens. As result, eight adults from *C*. *macropomum* and tambacu were selected, with both groups presenting a mean body length of 0.6 ± 0.2 m and weight of 3 ± 0.3 kg. The specimens were euthanized for the removal of fragments of the skin and muscle. The tissue samples were stored immediately in 1 mL of RNAlater (Ambion, Life Technologies, USA) at 4°C for 24 hours and then transferred to an ultrafreezer at -80°C. To ensure the identification of specimens (pure *vs*. hybrids), fragments of muscle were also stored in 100% ethanol at -20°C for DNA extraction.

It is important to note that the *C*. *macropomum* is a rheophilic species that is unable to breed in captivity [7]. These fish present obvious sexual dimorphism only during the breeding period, when the females acquire a soft, rounded abdomen, as well as a prominent, reddish urogenital papilla, while the male expel fluid semen after gentle abdominal massage [23]. As the specimens were collected during the non-breeding period, none of the individuals presented these characteristics, which impeded the differentiation of males and females.

### DNA extraction, PCRs and sequencing

The total DNA was isolated using phenol-chloroform and precipitation in ethanol [24]. The integrity of the DNA samples was verified by electrophoresis in 1% agarose gel stained with GelRed, followed by visualization under UV light.

The isolation and amplification of the mitochondrial Control Region (D-loop) was based on Polymerase Chain Reaction (PCR), as described by Gomes et al. [13]. The products of the amplification were sequenced using the Big Dye Terminator v. 3.1 Cycle Sequencing kit (Applied Biosystems), followed by electrophoresis in an automatic sequencer (Genetic Analyzer 3500). The sequences were then edited and aligned in BIOEDIT 7.2.5 [25]. The identification of the mitochondrial maternal lineage was based on comparisons with the sequences of the native species provided by Gomes et al. [13] and the sequences available in GenBank (*Colossoma macropomum* DQ480074 and *Piaractus mesopotamicus* AF283959).

### Multiplex PCR

The *C*. *macropomum* and hybrids individuals were also identified by multiplex PCR using the nuclear α-tropomiosin, as described by Gomes et al. [13]. We, then, used the universal primer Tropo Serra F (5’-GAGTTGGATCGGGCTCAG-3’) and species-specific primers (Tropo Cm R 5’-ATACAACAATGCCATCGCT-3’ and Tropo Pm R 5’-CTTCAGCTGGATCTCCTGA-3’).

### Extraction of the total RNA

The total RNA was isolated using the PureLink RNA mini kit (Ambion, Life Technologies, USA) according to the manufacturer’s instructions. Equal concentrations and volumes of the tissue sampled from different individuals were combined to produce four RNA pools, separated by organism and type of tissue. The samples were treated with RNAse-free DNAse (Invitrogen, CA, USA) to remove any DNA contaminants. The amount of RNA extracted was determined using a PicoDrop spectrophotometer (Picodrop, United Kingdom), and the quality, with a Bioanalyzer 2100 (Agilent Technologies). The integrity of the samples was confirmed by electrophoresis in 1.5% agarose gel. All the RNA samples were treated with RiboMinus (Invitrogen, CA, USA), according to the manufacturer’s instructions, for the selective depletion of the ribosomal RNA (rRNA) transcripts from the total RNA. The RNA samples were then stored at –80ºC prior to the pyrosequencing reaction.

### Construction of the cDNA library

Four *C*. *macropomum* and tambacu libraries were established. Complementary DNA (cDNA) libraries were constricted in two steps, firstly, by the fragmentation of the total RNA and then the synthesis of the double-stranded cDNA. The initial quantity of RNA used in the fragmentation was approximately 600 ng/μ, with zinc chloride (ZnCl2) and Tris-HCL being added to initiate the fragmentation process. The integrity of the fragments was verified in the Bioanalyzer 2100 (Agilent Technologies, Santa Clara, CA, USA), using the RNA 6000 Pico kit. The double strands of the cDNA were then obtained using the cDNA Synthesis System kit (Roche), with the following steps: denaturation of the RNA by the addition of a randomic primer, synthesis of the first cDNA strand, synthesis of the second cDNA strand, and the purification of the double cDNA strands. The irregular ends of the cDNA fragments generated by this process were then repaired. The cDNA fragments were quantified using a TBS 380 QuantiFluor fluorometer (Promega, USA), according to the manufacturer’s instructions. The size of the cDNA fragments was verified in a bioanalyzer (Agilent Technologies, Santa Clara, CA, USA) using the High Sensitivity DNA chip.

### Amplification of the cDNA library and sequencing

The cDNA fragments of all the samples (*C*. *macropomum* and tambacu) were pyrosequenced in the 454 GS FLX Titanium platform (Roche, Branford, CT, USA). The emulsion PCR (emPCR) was based on the enrichment, purification, and preparation of the Pico Titer Plate (PTP), conducted according to the manufacturer’s instructions. All the libraries were sequenced five times, with each run using a PTP with two regions. The raw sequence reads have been submitted in the National Center for Biotechnology Information (NCBI) Sequence Read Archive (SRA) database (Bioproject: PRJNA353388/ SRX2375124, SRX2375122, SRX2375120, SRX2375119).

### Computational analysis

#### Pre-processing

The raw Sff files were converted into FastQ in Geneious v. 7.1.7 [26], and the adapters were removed. The ribosomal reads were then filtered using the default parameters and compared with the database available in SortMeRNA v. 2.0 [27]. The subsequent non-rRNA reads were separated by size (100 bps), quality (>20) and homopolymers (<12) in Mothur v. 1.35.1 [28].

#### *De novo* assembly

The contigs were assembled in Newbler v. 3.0 [29] and Mira v. 4.0.2 [30]. Both packages adopt the overlap-layout-consensus (OLC) algorithm [31]. The contigs obtained for each tissue and each assembler were clustered in CD-HIT-EST v. 4.6.4 [32] to remove redundant elements based on a minimum overlapping length of 60 bps and minimal overlapping similarity of 95%. A reference transcriptome was generated from the entire set of assembled contigs.

#### Assessment of transcriptome assembly completeness

The BUSCO v3 (Benchmarking Universal Single-Copy Orthologs) program [33] was used to evaluate transcriptome completeness. The transcriptomes were compared with a predefined set of eukaryote and metazoan single-copy orthologs from the OrthoDB v9 database [34], assuming a cut-off of 1E-3. This analysis was based on the complete database of the transcripts of all the samples (skin and muscle) from both organisms.

#### Functional annotation

The contigs were compared with non-redundant protein sequences of the Swissprot protein (Swiss-Prot) dataset using BlastX, assuming a cut-off of 1E-3 and similarity of at least 30%. The Swiss-Prot is a highly accurate protein database with high rates of annotation, reduced redundancy, and high levels of integration with other datasets [35]. The XML outputs from BlastX were annotated in Blast2GO v. 3.3 [36], being assigned to one of the three main categories of Gene Ontology (GO): Biological Processes (BP), Molecular Function (MF), and Cell Components (CC). The contigs were then mapped according to their known metabolic and molecular pathways in the vertebrates, using the Kyoto Encyclopedia of Genes and Genomes (KEGG) [37].

#### Differential expression

The total reads of each library were mapped in STAR 2.5 [38] based on the reference transcriptome (the entire set of assembled contigs). The individual counts of the transcripts identified in each tissue were obtained in HTSeq 0.6.1 [39], and were used as input to estimate the abundance of transcripts in RPKM (Reads per Kilobase per Million mapped reads) in the R statistical package. The read counts were normalized using the RPKM function of the EdgeR package [40]. The online software IDEG6 (p value <0.001) [41] was used to identify the differentially expressed genes (DEGs), confirmed by Fold-change log 2 (Log2FC) values ≤ -1, ≥ 1. The Heatmap graph was based on the RPKM values of the DEGs, as identified by the IDEG6 (≤ 0.001) and Log2FC values. The CIM function of the mixOmics package [42] was used to run the correlation analysis, and all transcripts that presented read numbers of zero in at least one library were removed from the analysis. The Volcano Plot was based on the significant p (≤ 0.001) and Log2FC (≤ -1, ≥ 1) values. The heatmaps were produced in Heatmap.2 [43] and the Volcano plots were obtained in Ggplot [44] from the R package.

## Results and discussion

### Genetic identification of the organisms

The morphological similarities between the *C*. *macropomum* and its hybrid forms have long been an impediment on the precise identification of the species reared in tropical aquaculture. Therefore, genetic markers are required to ensure the reliable identification of specimens. The markers used in the present study were the mitochondrial D-loop sequences and the multiplex PCR of the nuclear α-Tropomiosin gene. The multiplex PCR is widely used as a highly effective, low-cost tool for the rapid identification of interspecific hybrids and parent species [13, 45].

**Fig 1:**
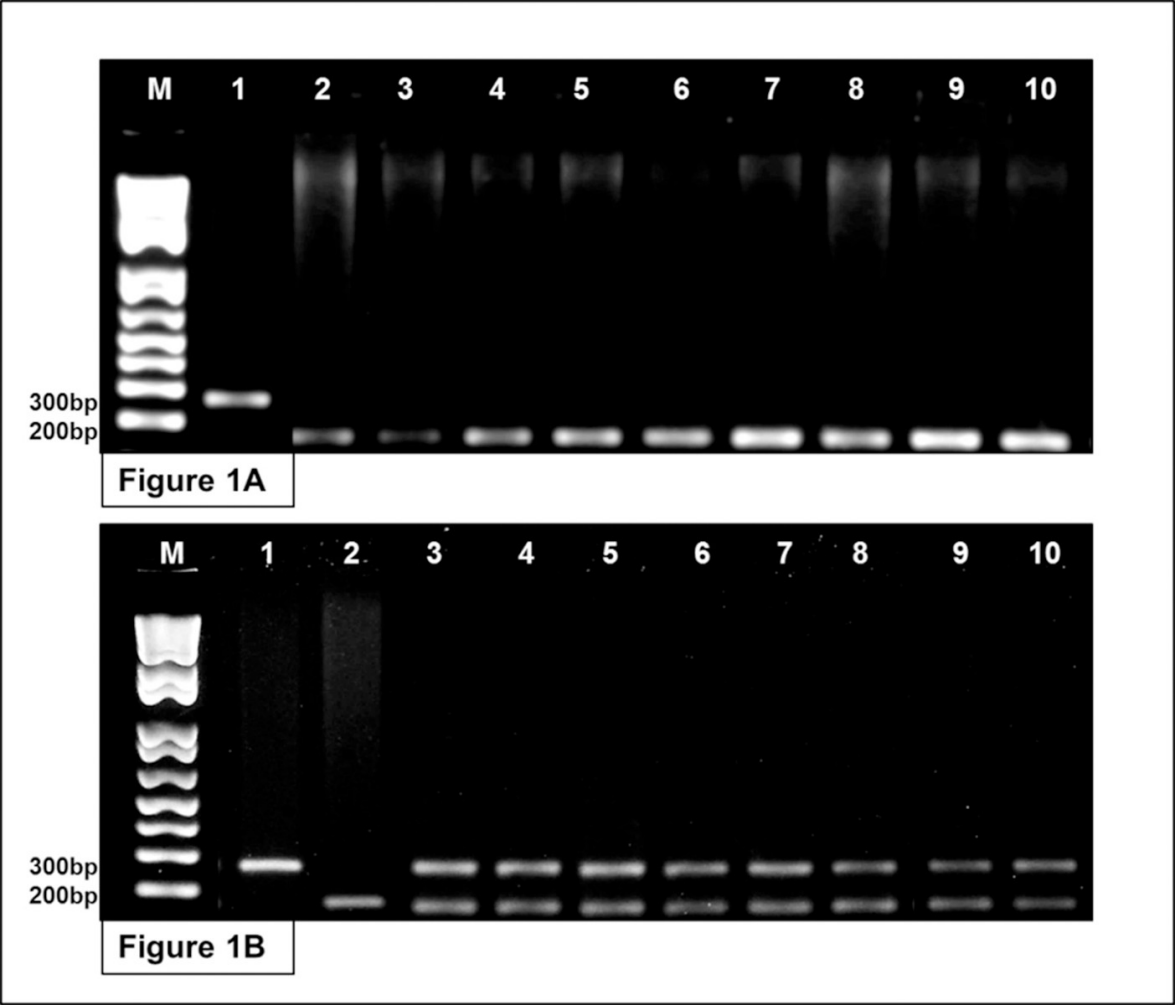
Molecular identification of the organisms based on multiplex PCR of α–tropomiosin gene. Fig 1A. Samples 1 and 2 represent the control species *P*. *mesopotamicus* (300 bps) and *C*. *macropomum* (200 bps), respectively. Samples 3–10 represent the pure *C*. *macropomum* individuals sampled in the present study. Fig 1B. Samples 1 and 2 represent the control species *P*. *mesopotamicus* (300 bps) and *C*. *macropomum* (200 bps), respectively. Samples 3–10 represent the hybrid tambacu individuals sampled in the present study.

As the mitochondrial DNA is inherited maternally in most eukaryotes [46, 47], the sequencing of the D-loop confirmed that the female parents of all the specimens analyzed were *C*. *macropomum* (S1 Table). The hybrids were discriminated from the *C*. *macropomum* by multiplex PCR, using the parental species (*C*. *macropomum* and *P*. *mesopotamicus*) as control. The results of the PCR yielded distinct band profiles for *P*. *mesopotamicus*, with fragments of 300 base pairs (bps), and *C*. *macropomum*, which presented bands of only 200 bps. The multiplex PCR produced a single band of 200 bps in eight of the 16 specimens analyzed, confirming that they belong to *C*. *macropomum* (Fig 1A). The other eight specimens had two distinct bands (one of 300 bps and the other of 200 bps), confirming their identification as tambacu hybrids. The 300-bp fragment is inherited from the *P*. *mesopotamicus* father and the 200-bp fragment from the *C*. *macropomum* mother (Fig 1B).

### Results of the sequencing and assembly

A total of 8,188,945 raw reads were obtained from the four libraries derived from the samples of the skin and muscle tissue of *C*. *macropomum* and the tambacu, using the Roche 454 platform (Table 1). After filtering, the remaining reads obtained from each type of tissue were assembled separately using MIRA and Newbler, providing a total of 572,760 contigs. The dataset was analyzed in CD-Hit-Est to remove redundant contigs and putative assembly errors, resulting in a final number of 400,845 contigs (Table 1). The use of two assemblers enriched both the number and the size of contigs, and is a successful, widely-used strategy [48, 49]. The contigs ranged in size from 200 to 49,961 bps. The largest contig was assembled from the *C*. *macropomum* muscle samples (S1 Fig). The N50 values for each type of tissue and other assembly metrics are shown in Table 1.

**Table 1:** *De novo* assembly and contigs obtained for each type of tissue in the *C*. *macropomum* and tambacu samples.

Organism	*C*. *macropomum* (Skin)	tambacu (Skin)	*C*. *macropomum* (Muscle)	tambacu (Muscle)
**Total Reads**	2,359,900	1,902,668	2,043,773	1,882,604
**Filtered Reads**	778909	430979	641023	843675
**Mean Quality**	32.4	32.82	30.74	31.08
**Average Length Reads**	442.5	363.54	409.82	401.16
***De novo* assembly**	**NEWBLER MIRA**	**NEWBLER MIRA**	**NEWBLER MIRA**	**NEWBLER MIRA**
**Assembled Reads**	225,669 276,699	57,499 77,521	282,652 251,282	329,908 336,303
**Total Contigs**	167,404 94,624	62,468 26,030	42,235 31,030	92,284 56,685
**Largest Contig**	16,759 10,530	7,664 5,909	49,960 22,203	43,182 49,465
**N50**	826 971	743 858	837 846	796 836
**Average Length**	817 910.5	753 776.8	833 768.5	817 786.8
**Contigs after CD-Hit-Est**	179,830	66,583	47,985	106,447
**Annotated Contigs**	18,200	7,932	11,316	20,875

### Assessment of the completeness of the transcriptome

The completeness of the transcriptome assembly of the *C*. *macropomum* and tambacu samples was assessed through comparison with the Benchmarking Universal Single-Copy Orthologs (BUSCO) for the metazoan and eukaryote gene sets. The genes sets were classified according to BUSCO parameters (Table 2) as either Complete (C), Single (S), Duplicated (D), Fragmented (F) or Missing (M). In this analysis, including the total set of transcripts of *C*. *macropomum* and the tambacu, 41.1% of the complete genes were observed in comparison with the metazoan gene set, and 42.3% in comparison with the eukaryote set. If the fragmented genes are included here, the percentage of genes recovered increases to 75.5% in comparison with the metazoans and 77.9% for the eukaryotes. On the other hand, 24.55% of the genes were classified as missing in comparison with the metazoan set, and 22.1% in comparison with the eukaryote set, which may reflect biological innovations in the *C*. *macropomum* and tambacu [33]. Although this cannot be evaluated due to the lack of data on the complete genome of these organisms, or even this result may be a consequence of problems in the data assembled.

**Table 2:** BUSCO statistics for the completeness of the *C*. *macropomum* and tambacu transcriptome assembly in comparison with the metazoan and eukaryote gene sets.

BUSCO	Metazoan Odb9 (%)	Eukaryote Odb9 (%)
**Complete**	41.1	42.3
**Single**	28.9	29.4
**Duplicated**	12.2	12.9
**Fragmented**	34.4	35.6
**Missing**	24.5	22.1

### Functional annotation

When the contigs from the present study were compared to the Swiss-Prot database using BlastX, 100,298 (25%) of the transcripts having significant hits. Of this total, 58,322 contigs were annotated (S2 Fig). Overall, 19.4% of the transcripts annotated in *C*. *macropomum* referred to the muscle, and 31.2% to the skin. In the tambacu 35.8% referred to the muscle and 13.8% to the skin. The top hits in BlastX revealed that *C*. *macropomum* and its hybrid shared transcripts with a number of other species of fish, amphibian, and mammal (S3 and S4 Figs).

### Gene Ontology (GO)

The Gene Oncology (GO) project provides a useful bioinformatics tool for the representation of genes or their products in different species and are especially informative for large datasets [50, 51]. In the present study, the GO analysis of the three principal domains, Biological Processes (BP), Molecular Function (MF), and Cell Components (CC), revealed a large number of functions for *C*. *macropomum* (15,634 GO terms) and tambacu (15,140 GO terms). The BP domain had the highest number of functions, with 10,491 (67.1% of the total) terms in the *C*. *macropomum* and 10,104 (66.7%) in the tambacu. This domain include a number of distinct systems, ranging from metabolic pathways to the physiological and behavioral features of an organism [52]. This domain was followed by MF, with 3,675 (23.5%) terms in the *C*.*macropomum* and 3,584 (23.7%) in the tambacu, while the CC had 1,469 (9.4%) in the *C*. *macropomum* and 1,452 (9.6%) in the tambacu (S5 Fig).

**Fig 2:**
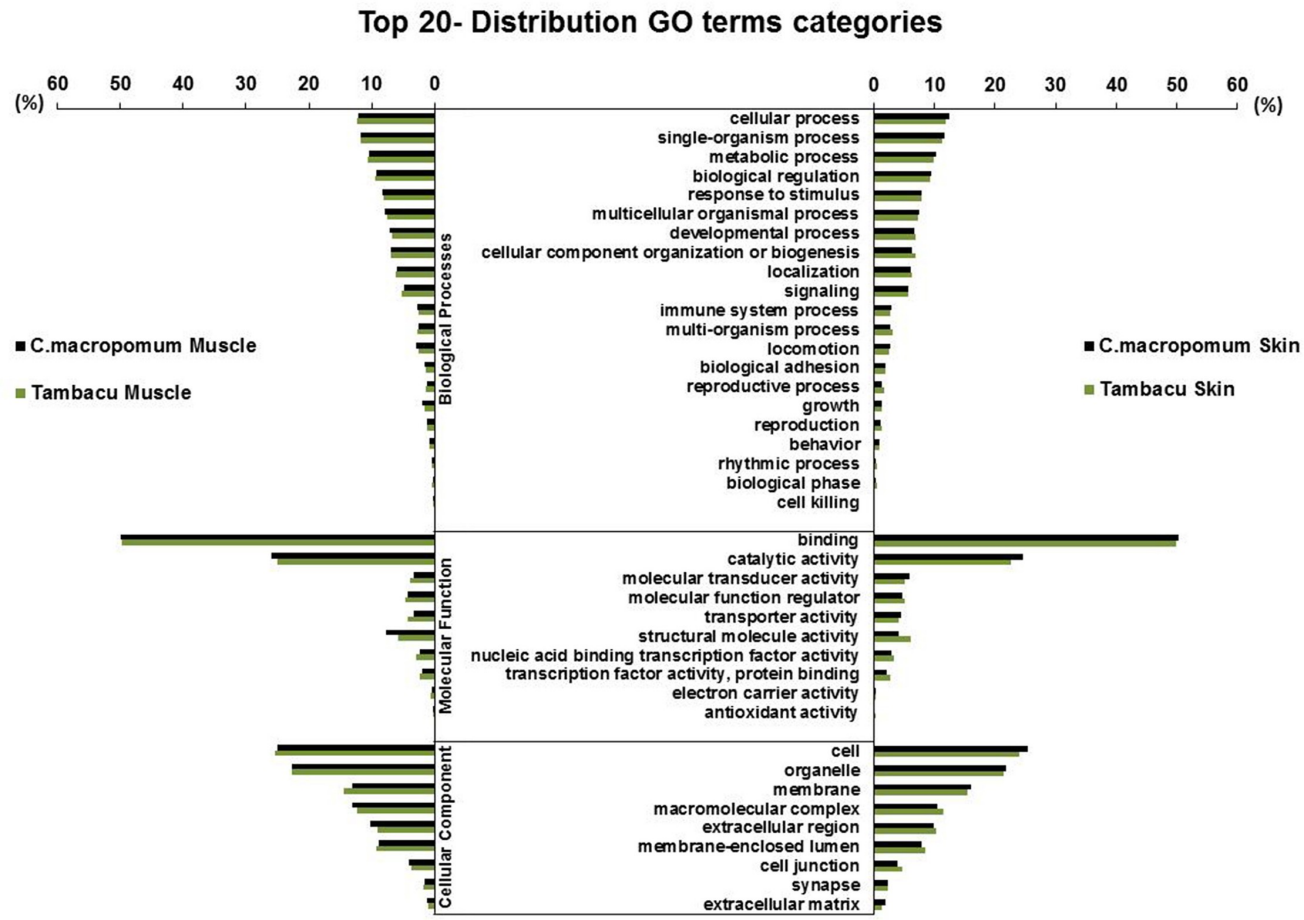
Top 20 GO terms. Processes with the highest number of transcripts within Biological Processes (BP), Molecular Function (MF) and Cell Components (CC) domains in the muscle and skin of *C*. *macropomum* and tambacu.

Similar results were obtained for the *C*. *macropomum* and tambacu in relation to the main functions observed in the BP, MF, and CC domains (Fig 2). The GO term “cellular process” (GO:0009987) was predominant in the BP domain (>12% in the four libraries), whereas in the MF domain, “binding” (GO:0005488) was the most dominant, with nearly half of the transcripts, followed by “catalytic activity” (GO:0003824), responsible for another quarter. In the case of the CC domain, the “cell” (GO:0005623) and “organelle” (GO:0043226) were the most common (45% of the transcripts in the four libraries). The distribution of the most expressed GO terms from each domain were similar to those reported in the transcriptomes of other vertebrate species [53–55].

### Kyoto Encyclopedia of Genes and Genomes (KEGG)

**Fig 3:**
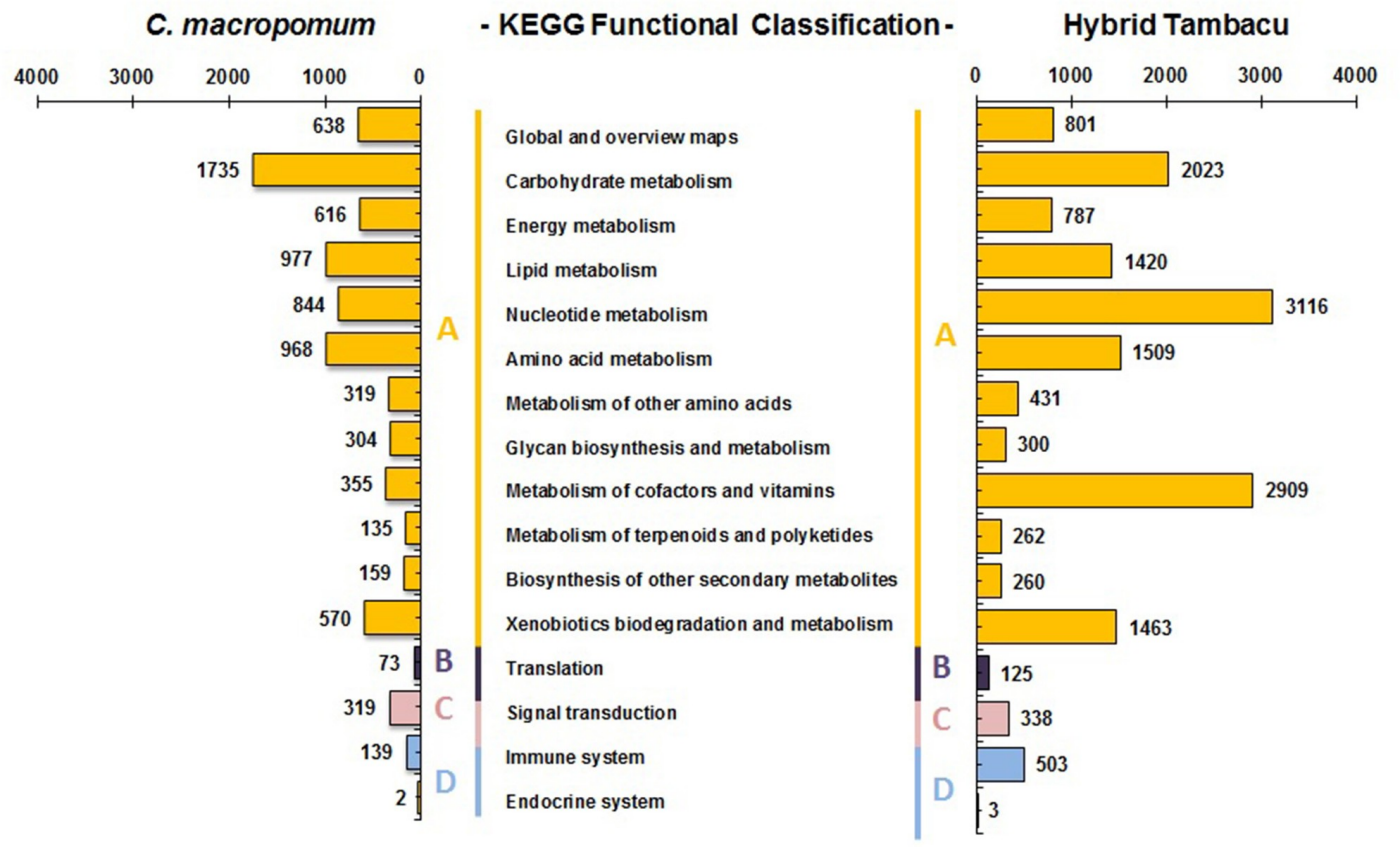
Pathway assignment based on the Kyoto Encyclopedia of Genes and Genomes (KEGG). (A) Metabolism categories, (B) Genetic information processing, (C) Environmental information processing, and (D) Organismal systems.

The KEGG analysis provides plots of the biochemical pathways, and is useful for the systematic analysis of gene function, focusing in particular on the gene and the molecular network [56]. In the present study, we combined the tissues from each organism, resulting in 132 signaling pathways for the *C*. *macropomum* and 133 for the tambacu, corresponding to 8276 (1337 enzymes) and 16,366 (1334 enzymes) sequences, respectively (S2 and S3 Tables). The pathways were classified in four categories: Metabolism, Genetic information processing, Environmental information processing, and Organismal system (Fig 3). The largest numbers of transcripts (7,620 in *C*. *macropomum* and 15,281 of those in the tambacu) were allocated to the Metabolism category. Within this category, a number of differences were found between the two organisms in the distribution of the subcategories. With 22.7% of the *C*. *macropomum* transcripts being involved with carbohydrate metabolism and 12.8% with lipid metabolism, whereas in the tambacu, 19.0% of the transcripts were involved with nucleotide metabolism and 17.7% with the metabolism of cofactors and vitamins.

We identified some differences in the most common pathways in the *C*. *macropomum* and tambacu, although in both organisms, the purine metabolism pathways were predominant, with 689 transcripts in the *C*. *macropomum*, and 2850 in the tambacu (S6 and S7 Figs). Previous reports on the fish transcriptome produced similar results, such as those recorded for the hybrid pufferfish (*Takifugu rubripes* x *Takifugu flavidus*) [57], for example, and the closely-related species, *Piaractus mesopotamicus* [6] and *Piaractus brachypomus* [58].

### Differential expression

The 454 FLX Genome sequencer (GS FLX) is suitable for the *de novo* sequencing of the transcriptome of non-model organisms [59–61], as confirmed in a number of previous studies [5, 6, 62]. As the organisms analyzed did not have a reference genome, we prepared a reference transcriptome, based on the entire set of assembled contigs. These sequences were mapped in the Star software, and counted in Htseq. Using rigorous parameters, we obtained a mapping index of 64.6% for *C*. *macropomum*, and 65.4% for the tambacu.

The present study provides the first comparative analysis of the transcriptomes of *C*. *macropomum* and the tambacu. Thus, based on the 400,845 contigs compiled in the four libraries, 21,986 genes were identified, including isophorms (S4 Table), with 16,632 being identified for *C*. *macropomum* and 14,835 for tambacu. The dataset from the muscle from both organisms comprised 13,951 genes, with 2,352 expressed only in the *C*. *macropomum* and 6,630 only in the tambacu. The dataset for the skin was composed of 16,857 genes, of which, 9,371 were expressed only in the *C*. *macropomum* and 2,772 only in the tambacu. The differences in the number of exclusive and shared genes support the hypothesis that the expressed transcripts depend on the development stage or genetic background of the sample specimens, given that genes may be expressed differentially in the same tissue [63, 64].

**Fig 4:**
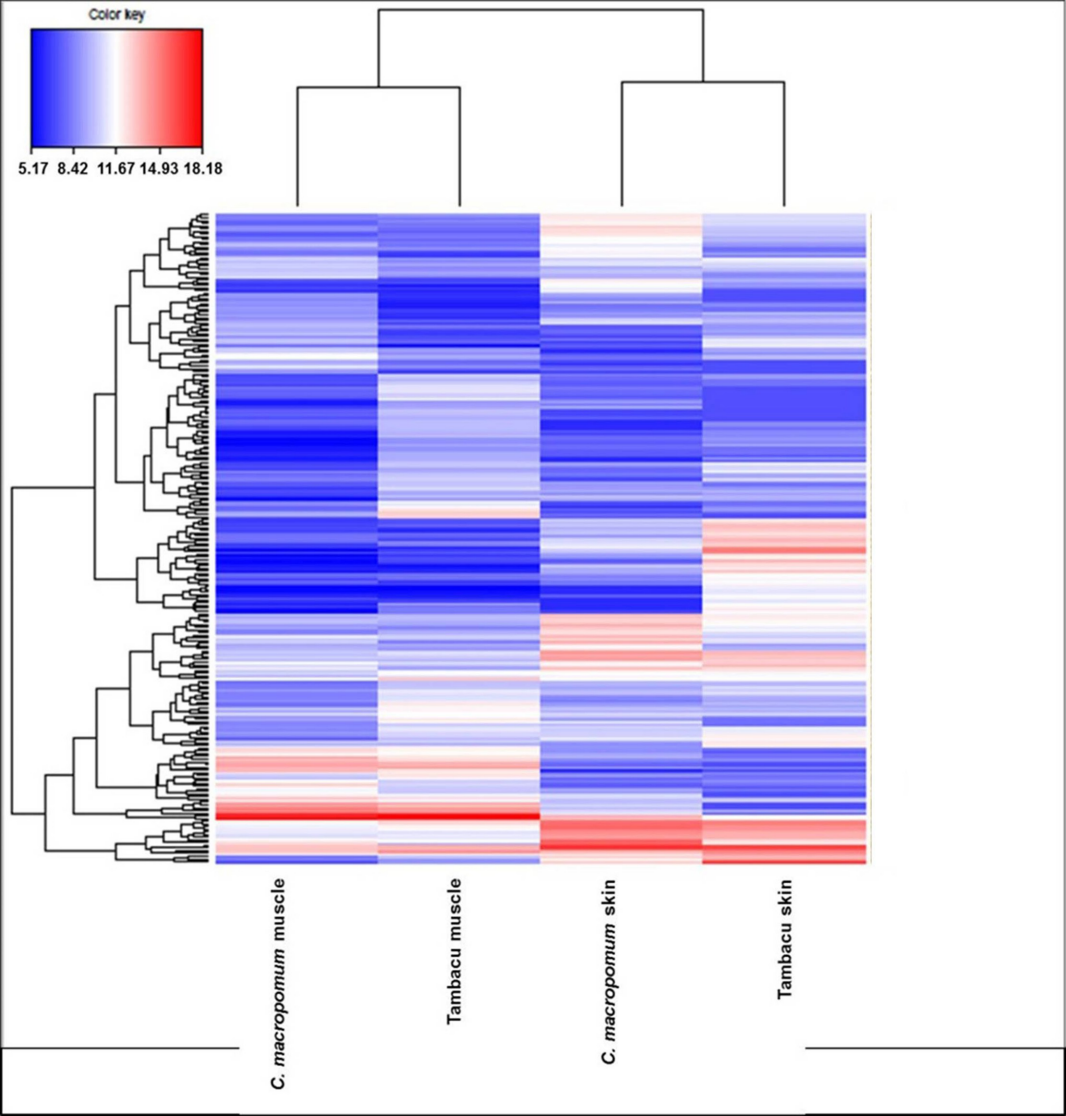
Heatmap graph. Constructed with RPKM values of differentially expressed genes for *C*. *macropomum* and tambacu (P Value ≤ 0,001; Log2FC ≥ 1 e ≤ -1).

**Fig 5:**
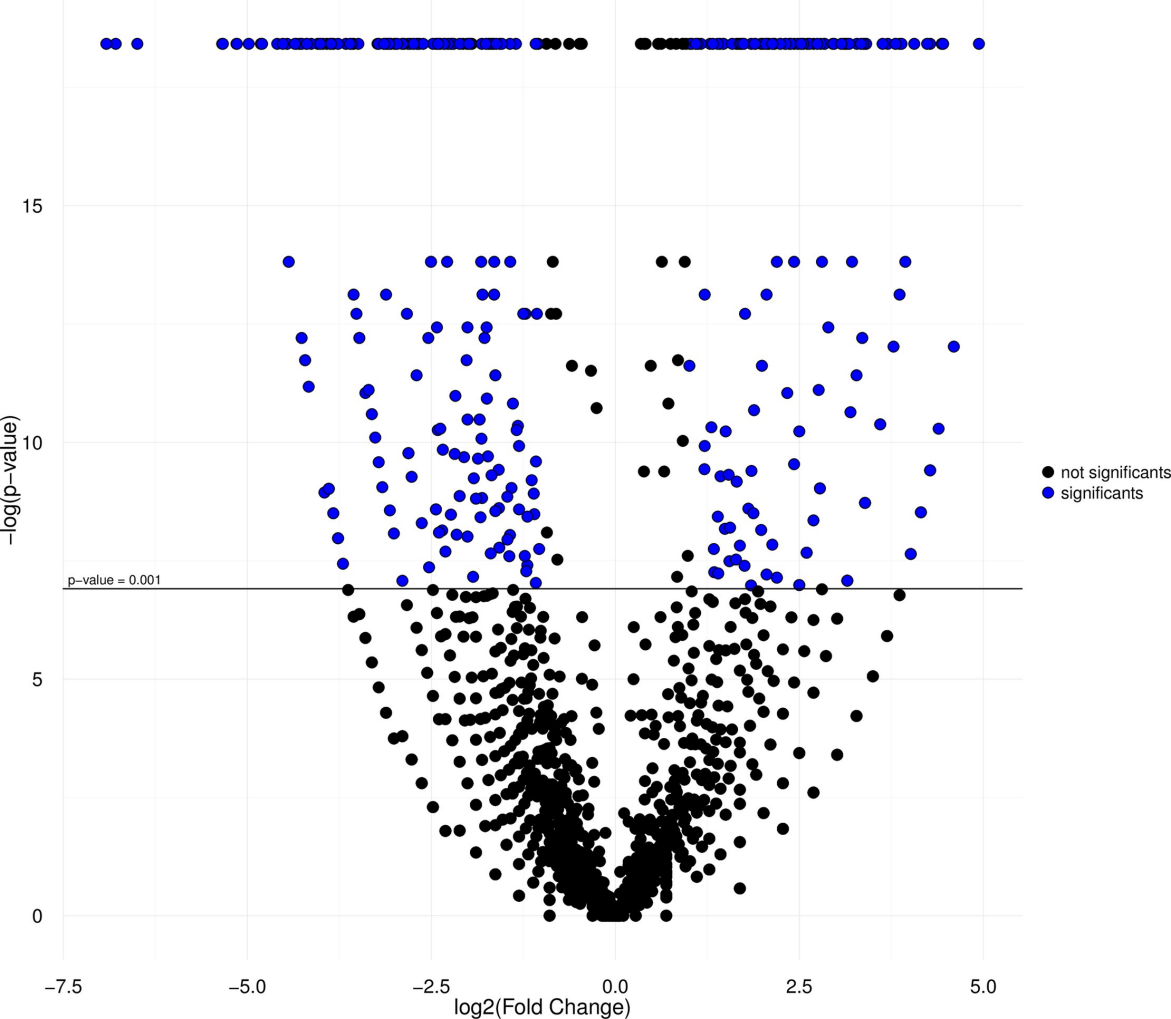
Volcano plot graph. Comparison of *C*. *macropomum* muscle DEGs (P ≤0,001; Log2FC ≥1) and tambacu muscle (P ≤ 0,001; Log2FC: ≤ -1), in red color the transcripts with significant values and in color blue the no significant.

**Fig 6:**
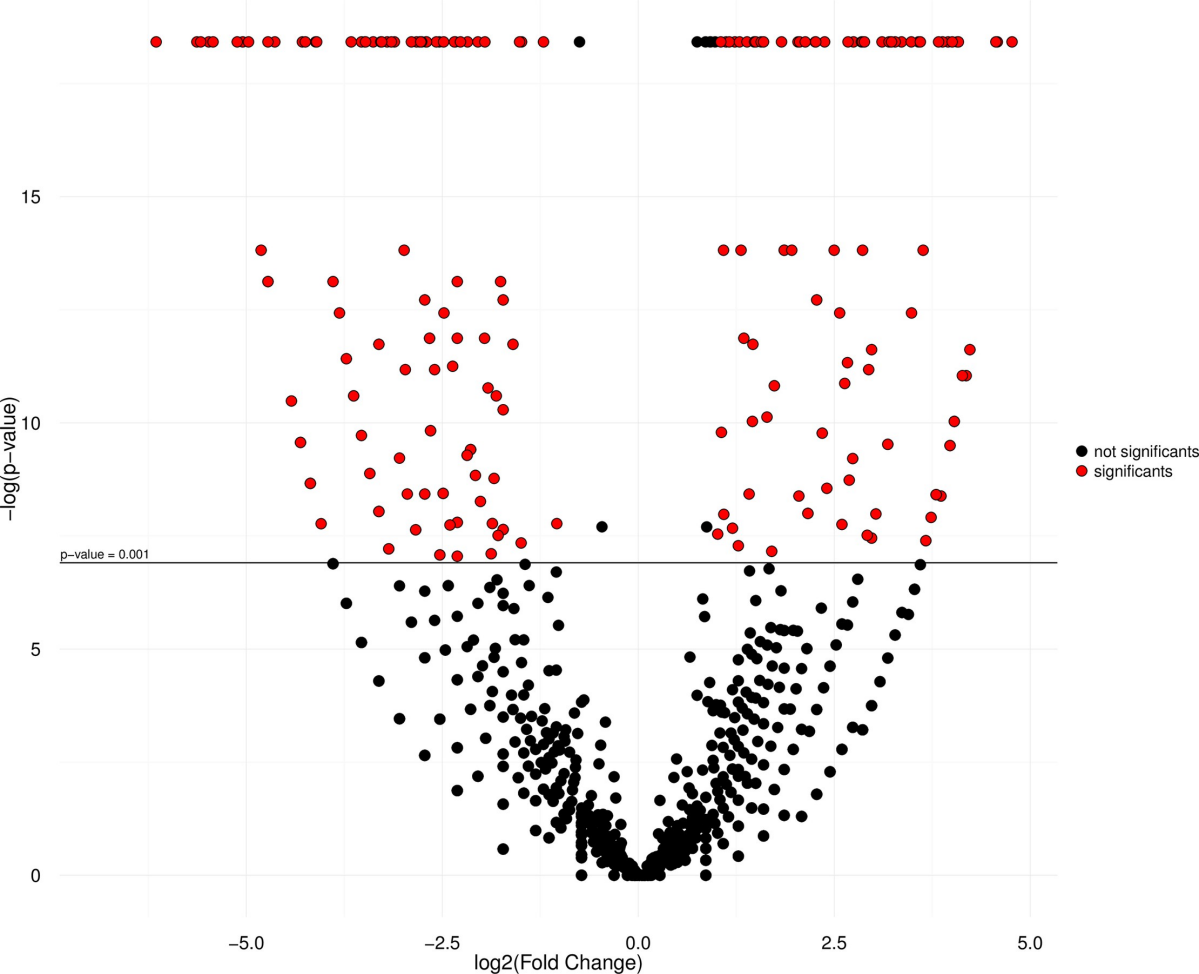
Volcano plot graph. Comparison of *C*. *macropomum* skin DEGs (P ≤0,001; Log2FC ≥1) with tambacu skin (P ≤ 0,001; Log2FC ≤ -1), in red color the transcripts with significant values and in color black the no significant.

The results of the heatmap analysis (Fig 4) indicate the clustering of tissues, with one clade formed by the muscle transcriptome of the *C*. *macropomum* and tambacu, and the other by the skin samples of the two organisms. The similar size of branches in both clades indicates equivalent patterns of divergence in both tissues in each organism. Each library was also characterized by a number of different groups of distinct and overexpressed genes, considering only the DEGs with RPKM <100 (all the genes included in the analysis are shown in S5 Table). The IDEG6 analysis (p < 0,001) together with the Log2FC values (≥ 1 and ≤ -1) revealed a number of differentially-expressed genes (see the DEGs in S6 Table). A total of 168 up-regulated genes were found in the library of the *C*. *macropomum* muscle, 222 in the tambacu muscle, 95 in the *C*. *macropomum* skin and 115 in the tambacu skin (Figs 5 and 6). These results reinforce the differences found in gene expression among organs, tissues or even different types of cell from the same organ [65].

### Genes expressed differentially in the muscle of the *C*. *macropomum* and tambacu

The largest contig with functional annotation (54,960 bps) was observed in the muscle of the *C*. *macropomum*, while the same transcript in the tambacu was slightly shorter, at 54,465 bps, but was nevertheless the most abundant protein (in reads) in this organism. This transcript refers to the gene involved in the synthesis of Titin (*ttn*), which is the largest and the most abundant protein in the skeletal and cardiac muscle of humans [66, 67]. In *C*. *macropomum*, the most expressed gene was the Myosin heavy chain fast skeletal muscle (*myss*), which is related to muscle contraction, and was also up-regulated in this species (Table 3).

**Table 3:** Some of the genes with the highest level of expression identified in the muscle and skin of *C*. *macropomum* and the tambacu.

Genes	Length	Over-Express	tissue	RPKM *C*.*macropomum*	RPKM tambacu	FC	Log2FC	P Value
**Myosin heavy chain, fast skeletal muscle**	7110	*C*. *macropomum*	Muscle	12424,47	5090,61	2,44	1,28	<0,001
**Parvalbumin beta**	2857	*C*. *macropomum*	Muscle	10822,37	832,01	13,007	3,71	<0,001
**Beta-enolase**	1650	*C*. *macropomum*	Muscle	3422,18	722,15	4,738	2,24	<0,001
**Actin, alpha skeletal muscle**	1135	*C*. *macropomum*	Muscle	1889,28	322,40	5,859	2,55	<0,001
**Myosin-2**	1379	*C*. *macropomum*	Muscle	768,64	35,08	21,905	4,45	<0,001
**ADP/ATP translocase 1**	1398	tambacu	Muscle	146,74	1213,59	0,12	-3,04	<0,001
**Elongation factor 1-alpha**	2219	tambacu	Muscle	356,60	962,20	0,37	-1,43	<0,001
**4F2 cell-surface antigen heavy chain**	2078	tambacu	Muscle	21,15	852,84	0,02	-5,33	<0,001
**Alpha-crystallin B chain**	2344	tambacu	Muscle	20,83	845,08	0,02	-5,34	<0,001
**Tripartite motif-containing protein 54**	1689	tambacu	Muscle	52,84	668,71	0,07	-3,66	<0,001
**LINE-1 retrotransposable element ORF2 protein**	3900	*C*.*macropomum*	Skin	558,90	256,92	2,17	1,12	<0,001
**Collagen alpha-1 (I) chain**	5865	*C*.*macropomum*	Skin	511,16	33,03	15,4	3,95	<0,001
**Collagen alpha-3 (VI) chain**	9460	*C*.*macropomum*	Skin	334,63	28,08	11,9	3,57	<0,001
**LINE-1 reverse transcriptase homolog**	3533	*C*.*macropomum*	Skin	307,53	117,51	2,61	1,38	<0,001
**Cation channel sperm-associated protein**	4478	*C*.*macropomum*	Skin	85,37	9,88	8,63	3,10	<0,001
**Keratin, type II cytoskeletal 8**	2829	tambacu	Skin	160,02	1473,49	0,11	-3,21	<0,001
**Keratin, type I cytoskeletal 13**	1687	tambacu	Skin	47,70	826,93	0,05	-4,11	<0,001
**Ictacalcin**	809	tambacu	Skin	111,91	650,07	0,17	-2,53	<0,001
**Desmoplakin**	2927	tambacu	Skin	18,33	455,80	0,04	-4,63	<0,001
**Keratin, type I cytoskeletal 50 kDa**	1992	tambacu	Skin	10,10	333,48	0,03	-5,04	<0,001

Of the 168 up-regulated genes in the muscle of *C*. *macropomum*, those of the myosin gene family are the most prominent, including Myosin-1 (*myh1*), Myosin-2 (*myh2*), Myosin-3 (*myh3*), Myosin-4 (*myh4*), Myosin-7 (*myh7*), Myosin-7B (*my7b*), Myosin-8 (*myh8*), and Myosin-13, *myh13* (Table 3, S6 Table). This gene family encodes motor proteins, which are responsible for the contraction mechanism, as well as being involved in several mechanochemical processes in eukaryotes [68, 69]. In addition to this gene family, Actin (*acts*/a*cta1*) was also expressed prominently in the muscle, and is particularly active in the eukaryote cell, where it has a synergistic effect with myosin in the control of mobility and muscle contraction [70], as well as the genes that encode Myomesin 1 (*myom1*) and Myomesin 2 (*myom2*), the main components of M-line [71] (Table 3, S6 Table). It is interesting to note that all these up-regulated genes in *C*. *macropomum* are related to muscular contraction. This may be related to the fact that this species is rheophilic, and needs to make an enormous effort to swim upstream, against the current, to mature sexually. The individuals raised in captivity (as those used in the present study) can to mature sexually, although the final stage of the spawning process does not occur, given the absence of the external factors necessary to trigger the reproductive process [7].

The genes responsible for the expression of catalytic proteins were also abundant in the DEGs of the muscle of the *C*. *macropomum*, including the enzymes Beta Enolase (*enob*/ *eno3*), Triosephosphate Isomerase B (*tpisb*/*tip1b*), NAD(P)H dehydrogenase (quinone) 1 (*nqo1*), Enolase (*eno*), and Phosphoglucomutase-1 (*pgm1*), most of which are related to the synthesis or degradation of glucose and the metabolism of carbohydrates [72–74] (Table 3, S6 Table). This finding may account for the predominance of the carbohydrate metabolism subcategory identified in the KEGG analysis for *C*. *macropomum*.

The results obtained for the transcriptome of the tambacu muscle included several up-regulated genes related to processes such as aminoacid metabolism, stress, and catalysis. In the KEGG analysis of the tambacu muscle, nucleotide metabolism was the most common subcategory (involving 19% of the transcripts) in the “metabolism” category. This finding can be accounted for by the presence of a number of differentially-expressed genes related to the metabolism of aminoacids, such as Elongation factor 1-alpha (*eef1a*), 4F2 cell-surface antigen heavy chain (*4f2*/ *slc3a2*), and Large neutral amino acids transporter small subunit 4 (*lat4/ slc43a2*) [75–77] (Table 3, S6 Table). The DEGs observed in the muscle of the tambacu also included genes of the ubiquitin family and similar genes, such as Ubiquitin-40S ribosomal protein S27a (*rps27a*), F-box only protein 32 (*fbxo32*), AN1-type zinc finger protein 5 (*zfand5*) (Table 3, S6 Table). These genes are involved primarily in the regulation of proteins [78].

Genes of the Heat shock protein (HSP) family were also prominent in the up-regulated genes of the muscle of the tambacu: Heat shock protein beta-1 (*hspb1*), Heat shock protein beta-11 (*hspb11*/*hspbb*), Heat shock 70 kDa protein (*hsp70*), Heat shock 70 kDa protein 1 (*hsp71*), Heat shock protein beta-6 (*hspb6*), Heat shock protein beta-8 (*hspb8*) (S6 Table). The expression of this protein family is related mainly to environmental and physiopathological stress [79, 80]. The Alpha-crystallin B (*cryab*) proteins are also prominent in this group (Table 3), being characterized by their presence in a number of different types of tissue, including the skeletal muscle [81, 82]. Like other HSPs, these proteins act as molecular chaperones to prevent the incorrect association and aggregation of the polypeptide chain [83, 84]. The presence of a number of HSPs with prominent over-expression in the muscle of tambacu is noteworthy, given that the expression of these genes is usually a response to stress factors, including environmental conditions and disease [79, 80, 85]. The marked expression of the genes related to these proteins potentially indicates environmental effects and adaptive processes in the hybrid tambacu. In fact, the HSPs are helpful for the repair of protein damages, given that over-expression is usually sufficient to protect the cell from exposure to stressful conditions, such as high temperatures [80, 86].

Some transcripts related to processes such as catalytic action, defense, and oxidative stress were also relatively common in the DEGs of the tambacu muscle, including AMP desaminase 3 (a*mpd3*), AMP desaminase 1 (a*mpd1*) Phosphoglycerate mutase 2 (*pgam2*), Sestrin-1 (*Sesn-1*), and Thioredoxin-interacting protein (*txnip*) [87–89] (S6 Table).

### Genes expressed differentially in the skin of the *C*. *macropomum* and tambacu

The skin is a complex type of tissue that plays a key role in sensorial activities, thermoregulation, hormone metabolism, and defense [20, 90, 91]. In addition to these functions, the morphological similarities between the *C*. *macropomum* and the tambacu also justifies the analysis of this tissue. Overall, 210 DEGs were identified in the skin of *C*. *macropomum* and tambacu.

In the skin of the *C*. *macropomum*, the transcripts with the largest number of mapped reads corresponded to the transposable elements: Transposable element Tcb1 transposase (*tcb1*), Transposable element Tc1 transposase (*tc1a*), and Transposable element Tcb2 transposase (*tcb2*). These elements are DNA segments capable of moving through the genome of a cell (“jumping genes”), thus affecting the evolution and genome composition of plants and animals, inducing processes ranging from pathologies to genetic differences in these organisms [92–94]. As in the muscle, the genes that encode collagen proteins were also found to be over-expressed in the skin of *C*. *macropomum*, including: Collagen alpha-1(I) chain (*co1a1*), Collagen alpha-3(VI) chain (*co6a3*) and Collagen alpha-2(I) chain (*co1a2*). These proteins are abundant in animals and are important for the molecular architecture and the shape and mechanical properties of tissues [95, 96].

Another important gene family, that should be mentioned, is the Cation channel sperm-associated protein (*catsper1/ctsr1* and *catsper3/ctsr3*). Despite presenting RPKM values lower than 100 (Table 3, S6 Table), this gene group was up-regulated in the skin of *C*. *macropomum*. However, the contigs equivalent to these transcripts were not assembled in the tambacu. The proteins encoded by this gene form a complex ion channel that permits the Ca^2+^ to enter the flagellum of the spermatozoa, which ensures their hyperactivation and plays a key role in male fertility [97]. The lack of this transcript in the tambacu may be related to the infertility or subfertility of these hybrids. Although some authors have observed that tambacu males produce small amounts of spermatozoa following abdominal pressure, which indicates that these fish can be crossed with their parent species (unpublished data), it remains unclear whether the tambacu is effectively fertile. In addition, the presence of up-regulated transcripts of *catsper1* and *catsper3* in the *C*. *macropomum* skin may represent a potential genetic marker for sex differentiation of the specie, which would be important, given the lack of sexual dimorphism in these fish during non-breeding periods, as occurred in the present study.

With regard to the differential expression of genes in the skin of the tambacu, the transcript with the largest number of mapped reads was related to the keratin, type II cytoskeletal 8 (*krt8*) protein, which was also present in the DEGs. In fact, other transcripts that encode proteins of this large keratin family (classes I and II) were also found in the DEGs of the tambacu skin (Table 3, S6 Table). The products of this gene family are structural proteins of the epithelial cells, which are expressed differentially according to the cell layer and tissue. The functions of these proteins have not yet been determined fully, but they are known to be involved in a number of processes, including structural support, cytoarchitecture, the response to stress, the regulation of signaling pathways related to apoptosis and protein synthesis, the distribution of organelles, and tissue repair and proliferation [98–100].

In contrast with the pattern observed in the *C*. *macropomum*, only a single gene of the collagen family–the collagen alpha-1(XVII) chain (*Col17a1*)–was observed in the DEGs of the tambacu skin. This transmembrane protein is a structural component of the hemidesmosomes in the epithelial cells and is associated with the keratinocytes [101]. One other important gene is the *dsp*, which encodes desmoplakin (Table 3). This is a key desmosome protein, which plays a major role in the attachment, assembly and stabilization of intermediate filaments, in addition to interacting with keratin proteins [102, 103].

Some authors have suggested that hybrids are more resistant and grow faster than their parent species [11, 104]. However, few studies have demonstrated any actual advantages in terms of increased productivity, and in fact, some data indicate that hybrids may be more susceptible to parasites [105, 106]. In the present study, major differences were found in the specificity and levels of gene expression in the muscle and skin of the *C*. *macropomum* and tambacu, which may contribute to the identification of the specific genes involved in the immunity, growth and fertility of these organisms. These findings raise a number of important questions that must be settled in order to confirm whether hybrids have any real advantages over their parent species, and whether they constitute a threat to the stocks of native species. We would further emphasize here the various up-regulated genes linked to stress found in the tambacu, which may be relevant to the farming of this hybrid, given that the stress of the captive environment may have a negative effect on the development of the fish. Given this, it will be very important to identify the factors that provoke this condition, not only for the organism itself, but also fish farming (the region in which this hybrid is farmed should also be evaluated).

## Conclusion

This is the first comparative analysis of the *C*. *macropomum* and its hybrid form, the tambacu. The results indicated that the NGS platform used in the study is a powerful tool for the identification of genes and molecular markers in non-model species, in addition to confirming that the combination of distinct assembly methods can enrich the dataset considerably. Despite of the lack of a reference genome, we obtained a satisfactory number of transcripts with functional annotation, associated typically with biological processes, with the skin presenting the greatest number of GO terms. The KEGG analysis also identified similarities in the metabolic pathways of the two organisms, with several transcripts in the metabolism category. There was a degree divergence in the number of exclusive genes, and the levels of gene expression and function between the *C*. *macropomum* and tambacu tissues. However, a significant number of genes remained uncharacterized, and are also of particular interest for future studies on the physiology, conservation, and genetic improvement of the native species, as well as the management of hybrid stocks. This research is thus an important contribution to the investigation of genes that contribute to potential advantages or disadvantages in the productivity of the hybrids, their implications for the conservation and management of *C*. *macropomum*, and provide a valuable tool for future research in functional genomics.

## Supporting information

S1 Fig Distribution of the transcript lengths recorded in the *C*. *macropomum* and the tambacu. (TIF)

S2 Fig Distribution of the data based on the results obtained in Blast2GO. (TIF)

S3 Fig Top-hit species distribution of the BlastX matches of *Colossoma macropomum* with the Swiss-Prot protein database. (TIF)

S4 Fig Top-hit species distribution of the BlastX matches of the tambacu with the Swiss-Prot protein database. (TIF)

S5 Fig The Gene Ontology (GO) analysis. Comparative distribution of the GO terms and the numbers of transcripts involved in Biological Processes (BP), Molecular Function (MF), and Cell Components (CC) in the *C*. *macropomum* and tambacu.
(TIF)

S6 Fig The functional KEGG classification of the transcripts of the muscle and skin tissue of *Colossoma macropomum*. (TIF)

S7 Fig The functional KEGG classification of the transcripts of the muscle and skin tissue of the tambacu. (TIF)

S1 Table Alignment of the mitochondrial DNA sequences of the individuals (*C*. *macropomum* and tambacu) sequenced in the present study. (XLSX)

S2 Table The KEGG data for the muscle and skin tissue of *Colossoma macropomum*. (XLSX)

S3 Table The KEGG data for the muscle and skin tissue of the tambacu. (XLSX)

S4 Table All the genes identified in the *C*. *macropomum* and tambacu, with their respective RPKM and Log2FC values. (XLSX)

S5 Table All the genes included in the Heatmap analysis with their respective RPKM and significance values. (CSV)

S6 Table All the DEGs identified in the *C*. *macropomum* and tambacu, with their respective RPKM and significance values. (XLSX)

## References

[1] Morozova O, Marra MA. Applications of next-generation sequencing technologies in functional genomics. Genomics. 2008;92:255–264. 10.1016/j.ygeno.2008.07.001 18703132

[2] Morozova O, Hirst M, Marra MA. Applications of New Sequencing Technologies for Transcriptome Analysis. Annu Rev Genomics Hum Genet. 2009;10:135–151. 10.1146/annurev-genom-082908-145957 19715439

[3] Datta S, Nettleton D. Stattisticall Analysis of Next Generation Sequencing Data. Springer: New York; 2014.

[4] Shen XY, Kwan HY, Thevasagayam NM, Prakki SRS, Kuznetsova IS, Ngoh SY, et al The first transcriptome and genetic linkage map for *Asian arowana*. Mol Ecol Resour. 2014;14:622–635. 10.1111/1755-0998.12212 24354690

[5] Thevasagayam NM, Sridatta PSR, Jiang J, Tong A, Saju JM, Kathiresan P, et al Transcriptome Survey of a Marine Food Fish: Asian Seabass (*Lates calcarifer*). Mar Sci Eng. 2015;3:382–400.

[6] Mastrochirico-Filho VA, Hata ME, Sato LS, Jorge PH, Forest F, Rodriguez MV, et al SNP discovery from liver transcriptome in the fish *Piaractus mesopotamicus*. Conserv Genet Resour. 2016;8:109–114.

[7] Lima CA, Goulding M. Os Frutos do Tambaqui: Ecologia, Conservação e Cultivo na Amazônia. Tefé, AM: Sociedade Civil de Mamiraua; 1998.

[8] Ministério da Pesca e Aquicultura. Boletim Estatístico da Pesca e Aquicultura 2011. Brasil: Brasília; 2014.

[9] Pinheiro MHP, Silva JWB, Nobre MIS, Pinheiro FA. Cultivo do Híbrido Tambaqui, *Colossoma macropomum* CUVIER, 1818, com a Pirapitinga *C*. *brachypomum* Cuvier, 1818, na Densidade de 5.000 Peixes/ha. Ciênc Agron. 1991;22:77–87.

[10] Giuseppe M. Retrocruce de hembras híbridos (F1) (*Colossoma macopomum* X *Piaractus mesopotamicus*) com machos de lãs especies parentales. Mem Soc Ci Nat La Salle. 1994;54:9–13.

[11] Melo JSC, Pereira JA. Crescimento do híbrido Tambacu (Fêmea de *Colossoma macropomum* X Macho de *Piaractus mesopotamicus)* em criação intensiva. Boletim técnico CEPTA. 1994;7:59–75.

[12] Calcagnotto D, Almeida-Toledo LF, Bernardino G, Toledo-Filho SA. Biochemical genetic characterization of F1 reciprocal hybrids between neotropical pacu (*Piaractus mesopotamicus*) and tambaqui *(Colossoma macropomum*) reared in Brazil. Aquaculture. 1999;174:51–57.

[13] Gomes F, Schneider H, Barros C, Sampaio D, Hashimoto D, Porto-Foresti F, et al Innovative molecular approach to the identification of *Colossoma macropomum* and its hybrids. An Acad Bras Cienc. 2012;84:517–525. 22534749 10.1590/s0001-37652012005000025

[14] Prado-Lima M, Val AL. Transcriptomic Characterization of Tambaqui (*Colossoma macropomum*, Cuvier, 1818) Exposed to Three Climate Change Scenarios. PLoS ONE. 2016;11:e0152366 10.1371/journal.pone.0152366 27018790 PMC4809510

[15] Gomes F, watanabe L, Nozawa S, Oliveira L, Cardoso J, Vianez J, et al Identification and characterization of the expression profile of the microRNAs in the Amazon species Colossoma macropomum by next generation sequencing. Genomics. 2017;67–74. 10.1016/j.ygeno.2017.02.001 28192178

[16] Martínez JG, Machado VN, Caballero-Gaitán SJ, Santos MF, Alencar RM, Escobar MD, et al SNPs markers for the heavily overfished tambaqui *Colossoma macropomum*, a Neotropical fish, using next-generation sequencing-based de novo genotyping. Conserv Genet Resour. 2017;9:29–33.

[17] Nunes JS, Liu S, Pértille F, Perazza CA, Villela OS, De Almeida-Val VF, et al Large-scale SNP discovery and construction of a high-density genetic map of *Colossoma macropomum* through genotypingby- sequencing. Sci Rep. 2017;7:46112 10.1038/srep46112 28387238 PMC5384230

[18] Brown TA. Genomes 2nd edition Oxford: Department of Biomolecular Science; 2002.

[19] Goetz FW, MacKenzie S. Functional genomics with microarrays in fish biology and fisheries. Fish and Fish. 2008;9:378–395.

[20] Elliott DG. Integumentary system In: Ostrander GK, editors. The laboratory fish, 1st edn New York: Academic Press; 2000 p. 271–306.

[21] Micallef G, Bickerdike R, Reiff C, Fernandes JMO, Bowman AS, Martin SAM. Exploring the Transcriptome of Atlantic Salmon (*Salmo salar*) Skin, a Major Defense Organ. Mar Biotechnol. 2012;14:559–569. 10.1007/s10126-012-9447-2 22527268

[22] Palstra AP, Beltran S, Burgerhout E, Brittijn AS, Magnoni LJ, Henkel CV, et al Deep RNA Sequencing of the Skeletal Muscle Transcriptome in Swimming Fish. PLoS ONE. 2013;8:e53171 10.1371/journal.pone.0053171 23308156 PMC3540090

[23] Murgas LD, Drumond MM, Pereira GM, Felizardo VO. Cycle manipulation and reproductive efficiency in native species of freshwater fish. Rev Bras Reprod Anim Supl. 2009;6:70–76.

[24] Sambrook J, Russel DW. Molecular cloning: a laboratory manual. New York: Cold spring harbor laboratory press; 2001.

[25] Hall TA. BioEdit: a user-friendly biological sequence alignment editor and analysis program for Windows 95/98/NT. Nucleic Acids Symp Ser. 1999;41:95–98.

[26] Kearse M, Moir R, Wilson A, Stones-Havas S, Cheung M, Sturrock S, et al Geneious Basic: An integrated and extendable desktop software platform for the organization and analysis of sequence data. Bioinformatics. 2012;28:1647–1649. 10.1093/bioinformatics/bts199 22543367 PMC3371832

[27] Kopylova E, Noé L, Touzet H. SortMeRNA: Fast and accurate filtering of ribosomal RNAs in metatranscriptomic data. Bioinformatics. 2012;1:1–7. 10.1093/bioinformatics/bts611 23071270

[28] Schloss PD, Westcott SL, Ryabin T, Hall JR, Hartmann M, Hollister EB, et al Introducing mothur: open-source, platformindependent, community-supported software for describing and comparing microbial communities. Appl Environ Microbiol. 2009;75:7537–7541. 10.1128/AEM.01541-09 19801464 PMC2786419

[29] Margulies M, Egholm M, Altman WE, Attiya S, Bader JS, Bemben LA, et al Genome sequencing in microfabricated high-density picolitre reactors. Nature. 2005;437:376–380. 10.1038/nature03959 16056220 PMC1464427

[30] Chevreux B, Pfisterer T, Drescher B, Driesel AJ, Müller WE, Wetter T, et al Using the miraEST Assembler for Reliable and Automated mRNA Transcript Assembly and SNP Detection in Sequenced ESTs. Genome Res. 2004;14:1147–1159. 10.1101/gr.1917404 15140833 PMC419793

[31] Miller JR, Koren S, Sutton G. Assembly algorithms for next-generation sequencing data. Genomics. 2010;95:315–27. 10.1016/j.ygeno.2010.03.001 20211242 PMC2874646

[32] Fu L, Niu B, Zhu Z, Wu S, Li W. CD-HIT: Accelerated for clustering the next-generation sequencing data. Bioinformatics. 2012;28:3150–3152. 10.1093/bioinformatics/bts565 23060610 PMC3516142

[33] Simão FA, Waterhouse RM, Ioannidis P, Kriventseva EV, Zdobnov EM. BUSCO: Assessing genome assembly and annotation completeness with single-copy orthologs. Bioinformatics. 2015;31(19):3210–2. 10.1093/bioinformatics/btv351 26059717

[34] Zdobnov EM, Tegenfeldt F, Kuznetsov D, Waterhouse RM, Simão FA, Ioannidis P, et al OrthoDB v9.1: cataloguing evolutionary and functional annotations for animal, fungal, plant, archaeal, bacterial and viral orthologs. OUP accepted manuscript. Nucleic Acids Res. 2016;45:1–15. 10.1093/nar/gkw1046 27899580 PMC5210582

[35] Bairoch A, Apweiller R. The Swiss-Prot protein sequence database and its supplement TrEMBL in 2000. Nucleic Acids Res. 2000;28:45–48. 10592178 10.1093/nar/28.1.45 PMC102476

[36] Gotz S, García-Gómez JM, Terol J, Williams TD, Nagaraj SH, Nueda MJ, et al High-throughput functional annotation and data mining with the blast2GO suíte. Nucleic Acids Res. 2008;36:3420–3435. 10.1093/nar/gkn176 18445632 PMC2425479

[37] Moriya Y, Itoh M, Okuda S. KAAS: Na automatic genome annotation and pathway reconstruction server. Nucleic Acids Res. 2007;35:W182–5. 10.1093/nar/gkm321 17526522 PMC1933193

[38] Dobin A, Davis CA, Schlesinger F, Drenkow J, Zaleski C, Jha S, et al STAR: ultrafast universal RNA-seq aligner. Bioinformatics. 2013;29:15–21. 10.1093/bioinformatics/bts635 23104886 PMC3530905

[39] Anders S, Pyl PT, Huber W. HTSeq- a Python framework to work with high-throughput sequencing data. Bioinformatics. 2014;1–4. 10.1093/bioinformatics/btu638 PMC4287950 25260700

[40] Robinson MD, McCarthy DJ, Smyth GK. EdgeR: a Bioconductor package for differential expression analysis of digital gene expression data. Bioinformatics. 2010;26:139–140. 10.1093/bioinformatics/btp616 19910308 PMC2796818

[41] Romualdi C, Bortoluzzi S, D'Alessi F, Danieli GA. IDEG6: a web tool for detection of differentially expressed genes in multiple tag sampling experiments. Physiol Genomics. 2003;12(2):159–62. 10.1152/physiolgenomics.00096.2002 12429865

[42] González I, Cao KL, Davis MD, Déjean S. Insightful graphical outputs to explore relationships between two ‘omics’ data sets. BioData Min. 2013;5:19. 10.1186/1756-0381-5-19 PMC3630015 23148523

[43] Warnes GR, Bolker B, Bonebakker L, Gentleman R, Liaw WA, Lumley T, et al. gplots: Various R Programming Tools for Plotting Data. R package version 3.0.1. 2016.

[44] Wickham H. ggplot2: Elegant Graphics for Data Analysis Springer-Verlag New York, 2009.

[45] Hashimoto DT, Mendonça FF, Senhorini JA, Jehud B, Oliveira C, Foresti F, et al Identification of hybrids between Neotropical fish *Leporinus macrocephalus* and *Leporinus elongates* by PCR-RFLP and multiplex-PCR: Tools for genetic monitoring in aquaculture. Aquaculture. 2010;298:346–349.

[46] Moritz C, Dowling TE, Brown WM. Evolution of animal mitochondrial DNA: relevance for population biology and systematic. *Ann Rev Ecol Syst*. 1987;18:269–292.

[47] Parker PG, Snow AA, Schug MD, Booton GC, Fuerst PA. What molecules can tell us about populations: choosing and using a molecular marker. Ecology. 1998;79:361–382.

[48] Kumar S, Blaxter ML. Comparing de novo assemblers for 454 transcriptome data. BMC Genomics. 2010;11:571 10.1186/1471-2164-11-571 20950480 PMC3091720

[49] Meyer B, Martini P, Biscontin A, Pitt C, Romualdi C, Teschke M, et al Pyrosequencing and de novo assembly of Antarctic krill (*Euphausia superba*) transcriptome to study the adaptability of krill to climate-induced environmental changes. Mol Ecol Resour. 2015;15:1460–1471. 10.1111/1755-0998.12408 25818178 PMC4672718

[50] Ashburner M, Ball CA, Blake JA, Botstein D, Butler H, Cherry JM, et al Gene Ontology: tool for the unification of biology. Nat Genet. 2000;25:25–29. 10.1038/75556 10802651 PMC3037419

[51] Conesa A, Gotz S, Garcia-Gomez JM, Terol J, Talon M, Robles M. Blast2GO: a universal tool for annotation, visualization and analysis in functional genomics research. Bioinformatics. 2005;21:3674–3676. 10.1093/bioinformatics/bti610 16081474

[52] Thomas PD, Mi H, Lewis S. Ontology annotation: mapping genomic regions to biological function. Curr Opin Cell Biol. 2007;11:4–11. 10.1016/j.cbpa.2006.11.039 17208035

[53] Salem M, Rexroad CE, Wang J, Thorgaard, Yao J. Characterization of the rainbow trout transcriptome using Sanger and 454-pyrosequencing approaches. BMC Genomics. 2010;11:564 10.1186/1471-2164-11-564 20942956 PMC3091713

[54] Jiang Y, Zhang S, Xu J, Feng J, Mahboob S, Al-Ghanim KA, et al Comparative Transcriptome Analysis Reveals the Genetic Basis of Skin Color Variation in Common Carp. PLoS ONE. 2014;9:e108200 10.1371/journal.pone.0108200 25255374 PMC4177847

[55] Deng T, Pang C, Lu X, Zhu P, Duan A, Tan Z, et al De Novo Transcriptome Assembly of the Chinese Swamp Buffalo by RNA Sequencing and SSR Marker Discovery. PLoS ONE. 2016;11(1):e0147132 10.1371/journal.pone.0147132 26766209 PMC4713091

[56] Ogata H, Goto S, Sato K, Fujibuchi W, Bono H, Kanehisa M. KEGG: Kyoto Encyclopedia of Genes and Genomes. Nucleic Acids Res. 1999;27:29–34. 9847135 10.1093/nar/27.1.29 PMC148090

[57] Gao Y, Zhang H, Gao Q, Wang L, Zhang F, Siva VS, et al Transcriptome Analysis of Artificial Hybrid Pufferfish Jiyan-1 and Its Parental Species: Implications for Pufferfish Heterosis. Plos One. 2013;8:e58453 10.1371/journal.pone.0058453 23520511 PMC3592836

[58] Jorge PH, Mastrochirico-Filho VA, Hata MA, Mendes NJ, Ariede RB, Freitas MV, et al Genetic Characterization of the Fish Piaractus brachypomus by Microsatellites Derived from Transcriptome Sequencing. Front Genet. 2018;9:46 10.3389/fgene.2018.00046 29520294 PMC5827183

[59] Vera C, Wheat CW, Fescemyer HW, Frilander MJ, Crawford DL, Hanski I, et al Rapid transcriptome characterization for a nonmodel organism using 454 pyrosequencing. Mol Ecol. 2008;17:1636–1647. 10.1111/j.1365-294X.2008.03666.x 18266620

[60] Meyer E, Aglyamova GA, Wang S, Buchanan-Carter J, Abrego D, Colbourne KJ, et al Sequencing and de novo analysis of a coral larval transcriptome using 454 GSFlx. BMC Genomics. 2009;10:219 10.1186/1471-2164-10-219 19435504 PMC2689275

[61] O'Neil ST, Dzurisin JDK, Carmichael RD, Lobo NF, Emrich SJ, Hellmann JJ. Population-level transcriptome sequencing of nonmodel organisms *Erynnis propertius* and *Papilio zelicaon*. BMC Genomics. 2010;11:310 10.1186/1471-2164-11-310 20478048 PMC2887415

[62] Hu Y, Huang M, Wang W, Guan J, Kong J. Characterization of gonadal transcriptomes from the turbot (*Scophthalmus maximus*). Genome. 2016;59:1–10. 10.1139/gen-2014-0190 26745327

[63] Adams J. Transcriptome: Connecting the genome to gene function. Nature Education. 2008;1:195.

[64] Cao D, Kocabas A, Ju Z, Karsi A, Li P, Patterson A, et al Transcriptome of channel catfish (*Ictalurus punctatus*): initial analysis of genes and expression profiles of the head kidney. Anim Genet. 2011;32:169–188. 10.1046/j.1365-2052.2001.00753.x 11531695

[65] Alvarez M, Schrey AW, Richards CL. Ten years of transcriptomics in wild populations: what have we learned about their ecology and evolution? Mol Ecol. 2015;24:710–725. 10.1111/mec.13055 25604587

[66] Bang ML, Centner T, Fornoff F, Geach AJ, Gotthardt M, McNabb M, et al The Complete Gene Sequence of Titin, Expression of an Unusual -700-kDa Titin Isoform, and Its Interaction with obscurin Identify a Novel Z-Line to I-Band Linking System. Circ Res. 2001;89:1065–1072. 11717165 10.1161/hh2301.100981

[67] Sanger JW, Sanger JM. Fishing out proteins that bind to titin. J Cell Biol. 2001;154:21–24. 10.1083/jcb.200106072 11448986 PMC2196855

[68] Hirayama Y, Watabe S. Structural differences in the crossbridge head of temperature associated myosin subfragment-1 isoforms from carp fast skeletal muscle. Eur J Biochem. 1997;246:380–387. 9208928 10.1111/j.1432-1033.1997.t01-2-00380.x

[69] Berg J, Powell BC, Cheney RE. A Millennial Myosin Census. Mol Biol Cell. 2001;12:780–794. 10.1091/mbc.12.4.780 11294886 PMC32266

[70] Sparrow JC, Nowak KJ, Hayley JD, Beggs AH, Walgren-Pettersson C, Romero N, et al Muscle disease caused by mutations in the skeletal muscle alpha-actin gene (ACTA1). Neuromuscul Disord. 2003;13:519–531. 12921789 10.1016/s0960-8966(03)00101-9

[71] Porter JD, Merriam AP, Gong B, Kasturi S, Zhou X, Hauser KF, et al Postnatal suppression of myomesin, muscle creatine kinase and the M-line in rat extraocular muscle. J Exp Biol. 2003;206:3101–3112. 12878677 10.1242/jeb.00511

[72] Davenport RC, Bash PA, Seaton BA, Karplus M, Petsko GA, Ringe D. Structure of the Triosephosphate Isomerase Phosphoglycolohydroxamate Complex: Na Analogue of the Intermediate on the Reaction Pathway. Biochemistry. 1991;30:5821–5826. 2043623 10.1021/bi00238a002

[73] Pancholi V. Multifunctional *a*-enolase: its role in diseases. Cell Mol Life Sci. 2001;58:902–920. 11497239 10.1007/PL00000910 PMC11337373

[74] StojKovic T, Vissing J, Petit F, Piraud M, Orngreen MC, Andersen G, et al Muscle Glycogenosis Due to Phosphoglucomutase 1 Deficiency. N Engl J Med. 2009;361:425–427. 10.1056/NEJMc0901158 19625727

[75] Condeelis J. Elongation factor 1α, translation and the cytoskeleton. Trends Biochem Sci. 1995;20:169–170. 7610475 10.1016/s0968-0004(00)88998-7

[76] Kanai Y, Segawa H, Miyamoto KI, Uchino H, Takeda E, Endou H. Expression Cloning and Characterization of a Transporter for Large Neutral Amino Acids Activated by the Heavy Chain of 4F2 Antigen (CD98). J Biol Chem. 1998;273:23629–23632. 9726963 10.1074/jbc.273.37.23629

[77] Guetg A, Mariotta L, Bock L, Herzog B, Fingerhut R, Camargo SMR, et al Essential amino acid transporter Lat4 (*Slc43a2*) is required for mouse development. J Physiol. 2015;593:1273–1289. 10.1113/jphysiol.2014.283960 25480797 PMC4358684

[78] Pickart CM, Eddins MJ. Ubiquitin: structures, functions, mechanisms. Biochim Biophys Acta. 2004;1695:55–72. 10.1016/j.bbamcr.2004.09.019 15571809

[79] Morimoto RI. Cells in stress: transcriptional activation of heat shock genes. Science. 1993;259:1409–1410. 8451637 10.1126/science.8451637

[80] Morimoto RI. Regulation of the heat shock transcriptional response: cross talk between a family of heat shock factors, molecular chaperones, and negative regulators. Genes Dev. 1998;12:3788–3796. 9869631 10.1101/gad.12.24.3788

[81] Dubin RA, Wawrousek EF, Piatigorsky J. Expression of the Murine αB-Crystallin Gene Is Not Restricted to the Lens. Mol Cell Biol. 1989;9:1083–1091. 2725488 10.1128/mcb.9.3.1083-1091.1989 PMC362698

[82] Jeanpierre C, Austruy E, Delattre O, Jones C, Junien C. Subregional physical mapping of an αB-crystallin sequence and of a new expressed sequence DllS877E to human llq. Mamm Genome. 1993;4:104–108. 8431633 10.1007/BF00290434

[83] Jong WW, Leunissen JAM, Voorter CEM. Evolution of the a-Crystallin / Small Heat-Shock Protein Family. Mol Biol Evol. 1993;10:103–126. 10.1093/oxfordjournals.molbev.a039992 8450753

[84] Derham BK, Harding JJ. α-Crystallin as a Molecular Chaperone. Prog Retin Eye Res. 1999;18:463–509. 10217480 10.1016/s1350-9462(98)00030-5

[85] Wu C. Heat Shock transcription factors: structure and regulation. Annu Rev Cell Dev. 1995;11:441–469. 10.1146/annurev.cb.11.110195.002301 8689565

[86] Parsell DA, Lindquist S. The function of heat-shock proteins in stress tolerance: degradation and reactiv ation of damaged proteins. Annu Rev Genet. 1993;27:437–496. 10.1146/annurev.ge.27.120193.002253 8122909

[87] Buckbinder L, Talbott R, Seizinger BR, Kley N. "Gene regulation by temperature-sensitive p53 mutants: identification of p53 response genes". Proc Nat Acad Sci U S A. 1994;91:10640–10644. 10.1073/pnas.91.22.10640 PMC45077 7938006

[88] Hancock CR, Brault JJ, Terjung RL. Protecting the cellular energy State during contractions: Role of Amp Deaminase. J Physiol Pharmacol. 2006;57:17–29. 17242488

[89] Zhou R, Tardivel A, Thorens B, Choi I, Tschopp J. Thioredoxin-interacting protein links oxidative stress to inflammasome activation. Nat Immunol. 2010;11:136–141. 10.1038/ni.1831 20023662

[90] Guellec DL, Morvan-Dubois G, Sire JY. Skin development in bony fish with particular emphasis on collagen deposition in the dermis of the zebrafish (*Danio rerio*). Int J Dev Biol. 2004;48:217–231. 10.1387/ijdb.031768dg 15272388

[91] Rakers S, Gebert M, Uppalapati S, Meyer W, Maderson P, Sell AF, et al ‘Fish matters’: the relevance of fish skin biology to investigative dermatology. Exp Dermatol. 2010;19:313–324. 10.1111/j.1600-0625.2009.01059.x 20158518

[92] Prak ET, Kazazian HH. Mobile elements and the human genome. Nat Rev Genet. 2000;1:134–144. 10.1038/35038572 11253653

[93] Biémont C, Vieira C. Junk DNA as an evolutionary force. Nature. 2006;443:521–524. 10.1038/443521a 17024082

[94] Venner S, Feschotte C, Biémont C. Transposable elements dynamics: toward a community ecology of the genome. Trends Genet. 2009;25:317–323. 10.1016/j.tig.2009.05.003 19540613 PMC2945704

[95] Kadler K. Extracellular matrix 1: Fibril-forming collagens. Protein Profile. 1995;2:491–619. 7584473

[96] Ricard-Blum S. The Collagen Family. Cold Spring Harb Perspect Biol. 2011;3:a004978 10.1101/cshperspect.a004978 21421911 PMC3003457

[97] Muratori M, Luconi M, Marchiani S, Forti G, Baldi E. Molecular markers of human sperm functions. Int J Androl. 2008;32:25–45. 10.1111/j.1365-2605.2008.00875.x 18298567

[98] Coulombe PA, Omary MB. ‘Hard’ and ‘soft’ principles defining the structure, function and regulation of keratin intermediate filaments. Curr Opin Cell Biol. 2002;14:110–122. 11792552 10.1016/s0955-0674(01)00301-5

[99] Gu LH, Coulombe PA. Keratin function in skin epithelia: A broadening palette with surprising shades. Curr Opin Cell Biol. 2007;19:13–23. 10.1016/j.ceb.2006.12.007 17178453

[100] Magin TM, Vijayaraj P, Leube RE. Structural and regulatory functions of keratins. Exp Cell Res. 2007;313:2021–2032. 10.1016/j.yexcr.2007.03.005 17434482

[101] Schäcke H, Schumann H, Hammami-Hauasli N, Raghunath M, Bruckner-Tuderman L. Two Forms of Collagen XVII in Keratinocytes a full-length transmembrane protein and a soluble ectodomain. J Biol Chem. 1998;273:25937–25943. 9748270 10.1074/jbc.273.40.25937

[102] Gallicano GI, Kouklis P, Bauer C, Yin M, Vasioukhin V, Degenstein L, et al Desmoplakin Is Required Early in Development for Assembly of Desmosomes and Cytoskeletal Linkage. J Cell Biol. 1998;143:2009–2022. 9864371 10.1083/jcb.143.7.2009 PMC2175222

[103] Fontao L, Favre B, Riou S, Geerts D, Jaunin F, Saurat JH, et al Interaction of the Bullous Pemphigoid Antigen 1 (BP230) and Desmoplakin with Intermediate Filaments Is Mediated by Distinct Sequences within Their COOH Terminus. Mol Biol Cell. 2003;14:1978–1992. 10.1091/mbc.E02-08-0548 12802069 PMC165091

[104] Martins ML, Onaka EM, Moraes FR, Bozzo FR, Paiva AMFC, Gonçalves A. Recent studies on parasitic infections of freshwater cultivated fish in the state of São Paulo, Brazil. Acta Sci Biol Sci. 2002;24:981985.

[105] Martins ML, Souza VN, Moraes JR, Moraes FR, Costa AJ. Comparative evaluation of the susceptibility of cultivated fishes to the natural infection with myxosporean parasites and tissue changes in the host. Braz J Biol. 1999;59:263–269.

[106] Tavares-Dias M, Neves LR, Santos EF, Dias MKR, Marinho RGB, Ono EA. *Perulernaea gamitanae* (Copepoda: Lernaeidae) parasitizing tambaqui (*Colossoma macropomum*) (Characidae) and the hybrids tambacu and tambatinga, cultured in northern Brazil. Arq Bras Med Vet Zootec. 2011;63:988–995.

