# Supplementary figures and images for: Comparative analysis of the transcriptome of the Amazonian fish species Colossoma macropomum (tambaqui) and hybrid tambacu by next generation sequencing
Source: PLoS One. 2019 Feb 25;14(2):e0212755. doi: 10.1371/journal.pone.0212755 (PMC6388931; doi:10.1371/journal.pone.0212755)

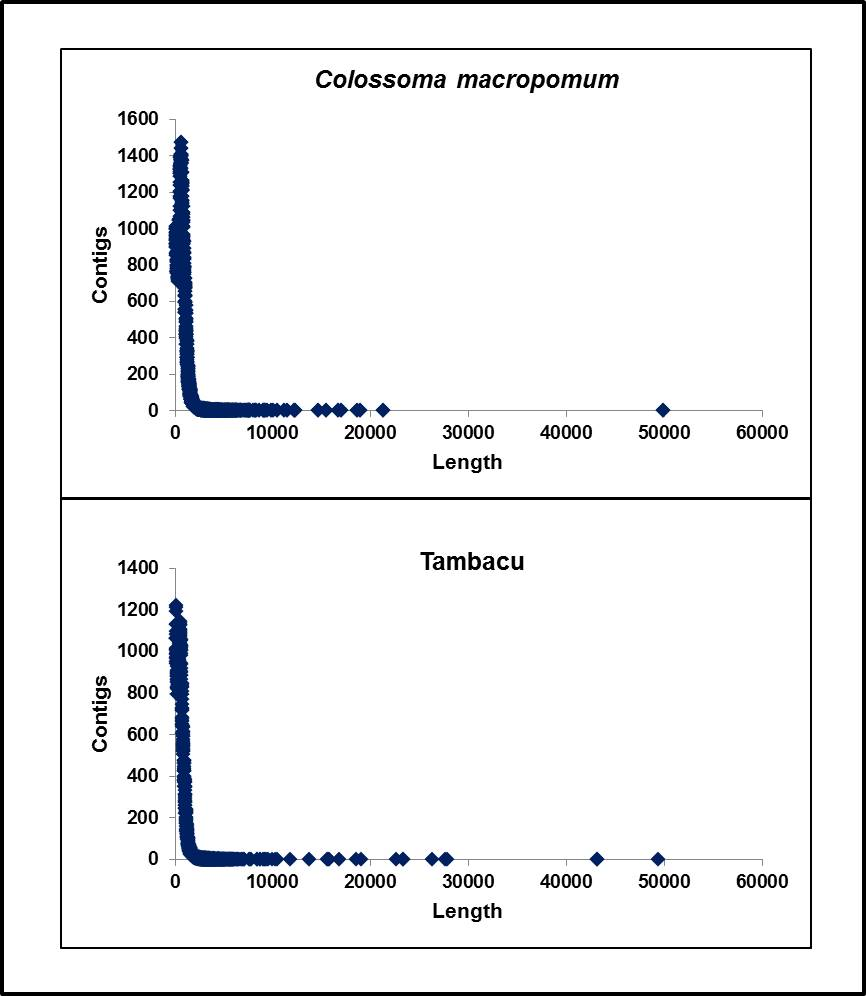

Supplement: S1 Fig — (TIF) [file pone.0212755.s001.tif]

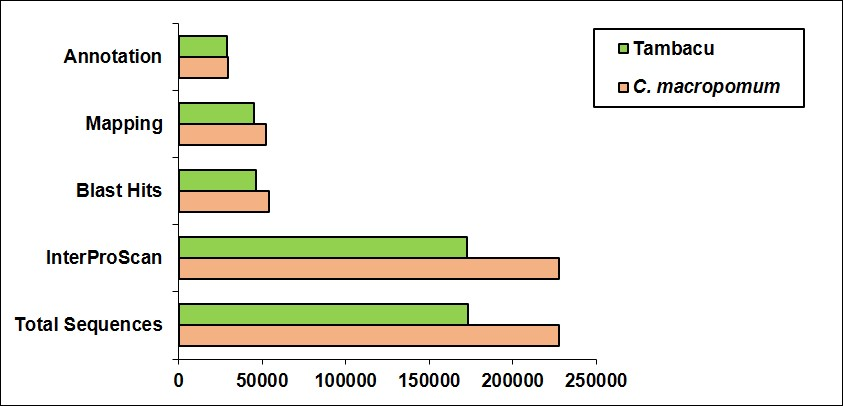

Supplement: S2 Fig — (TIF) [file pone.0212755.s002.tif]

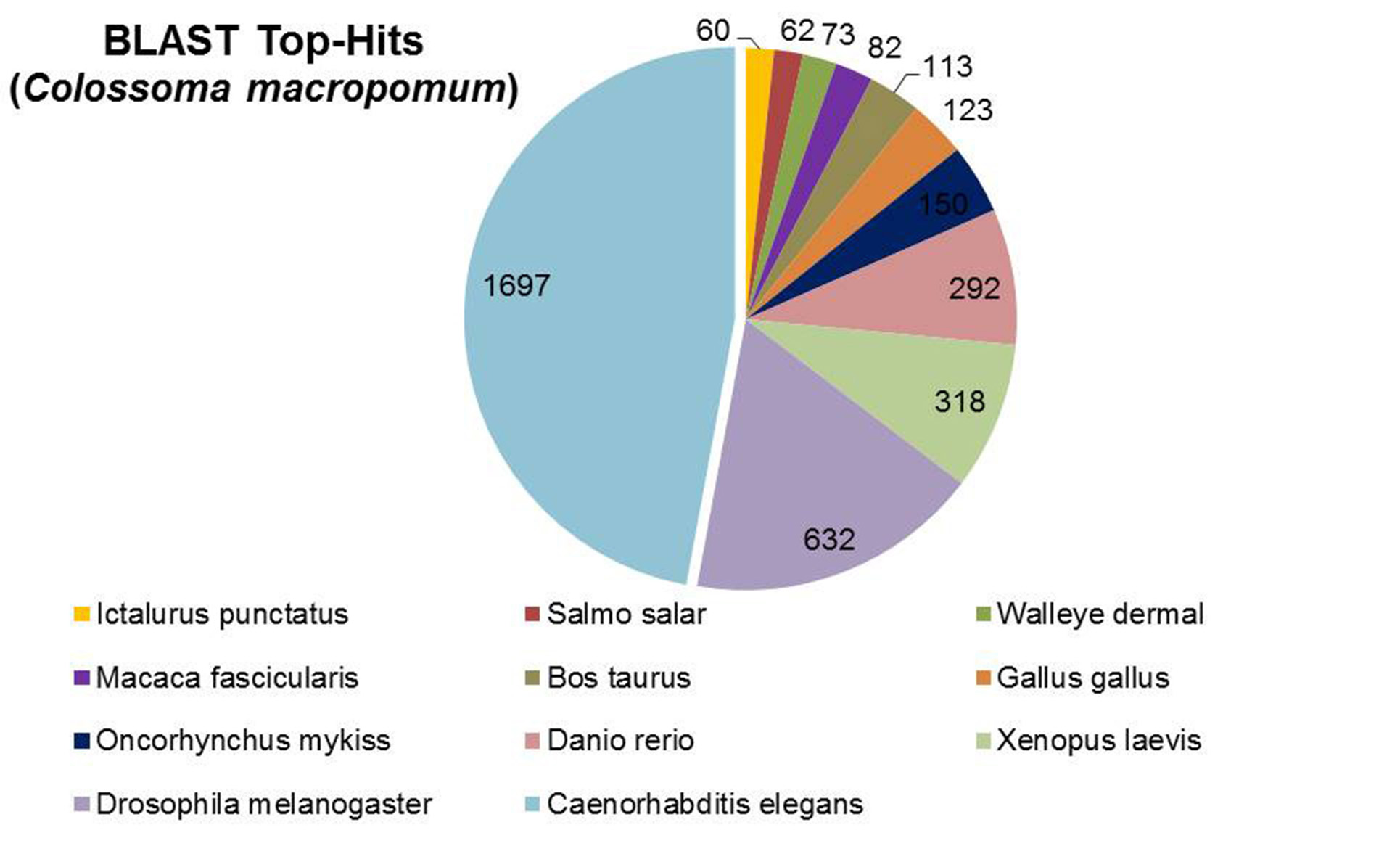

Supplement: S3 Fig — (TIF) [file pone.0212755.s003.tif]

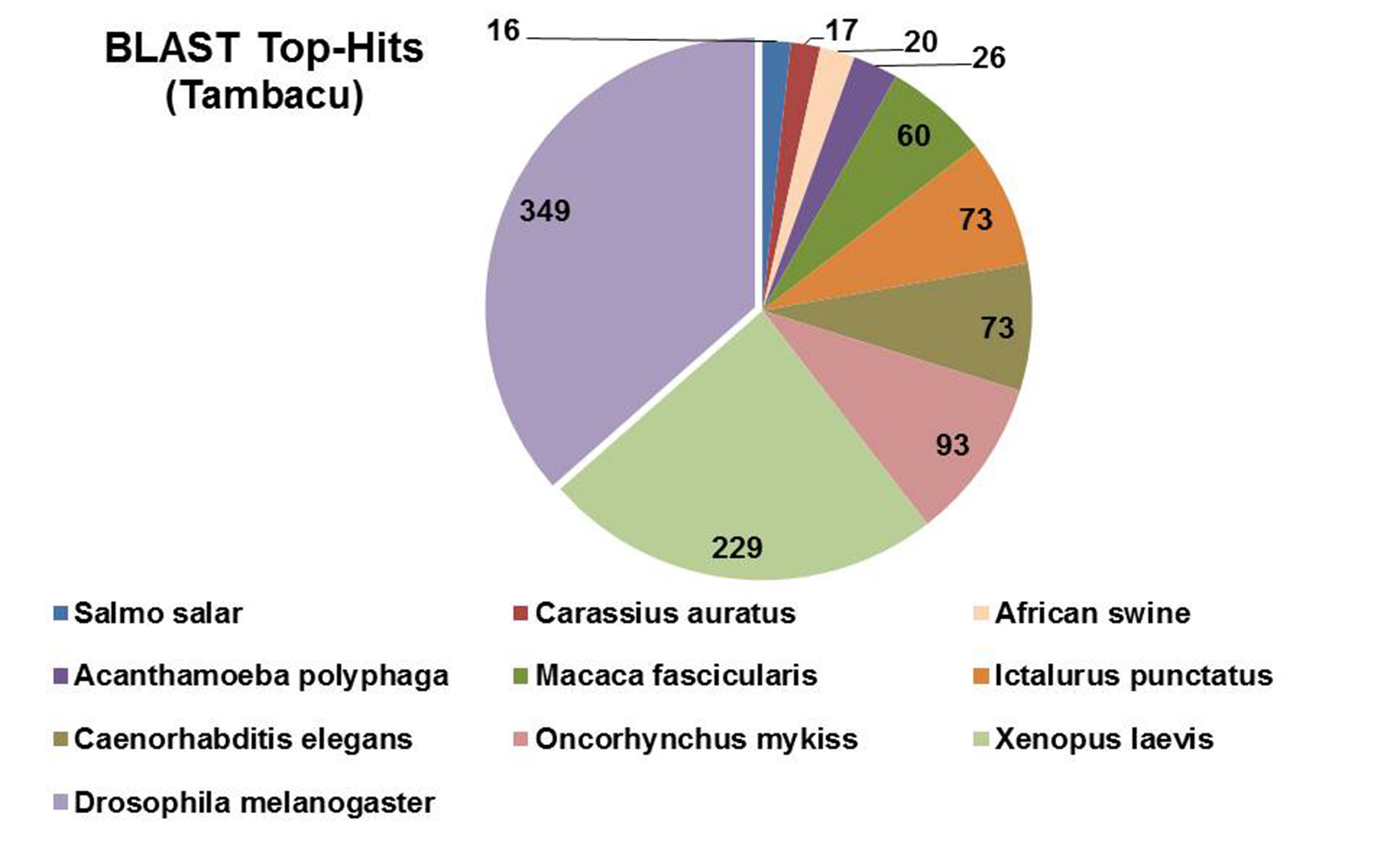

Supplement: S4 Fig — (TIF) [file pone.0212755.s004.tif]

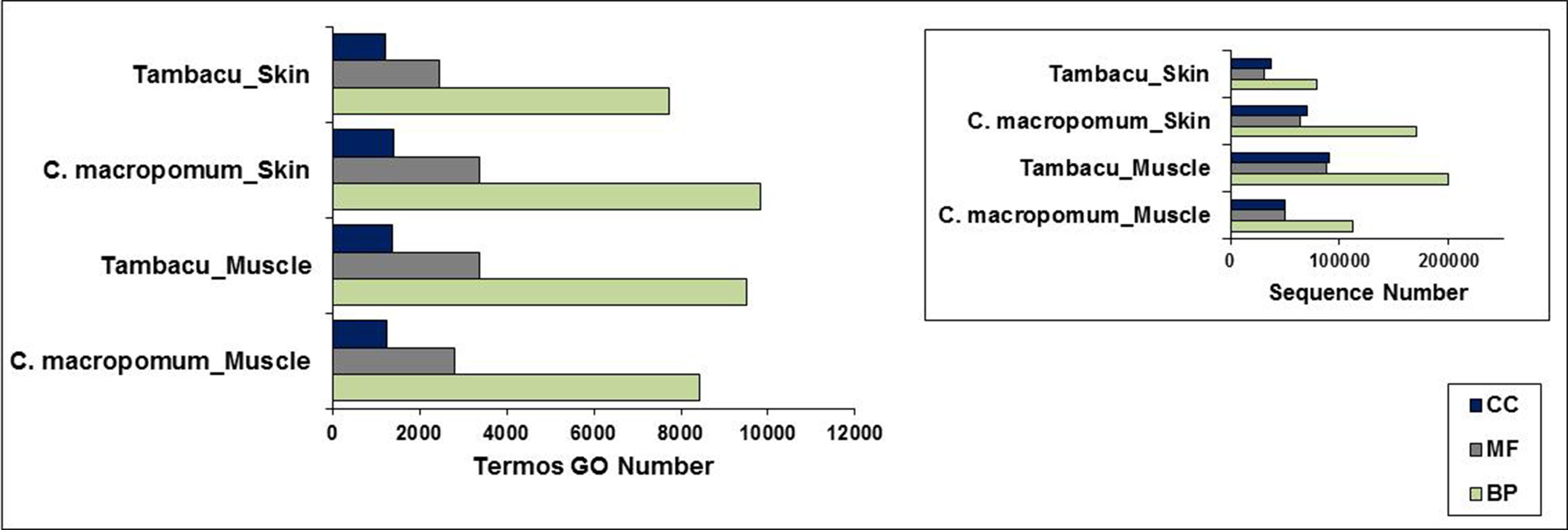

Supplement: S5 Fig — Comparative distribution of the GO terms and the numbers of transcripts involved in Biological Processes (BP), Molecular Function (MF), and Cell Components (CC) in the C. macropomum and tambacu. (TIF) [file pone.0212755.s005.tif]

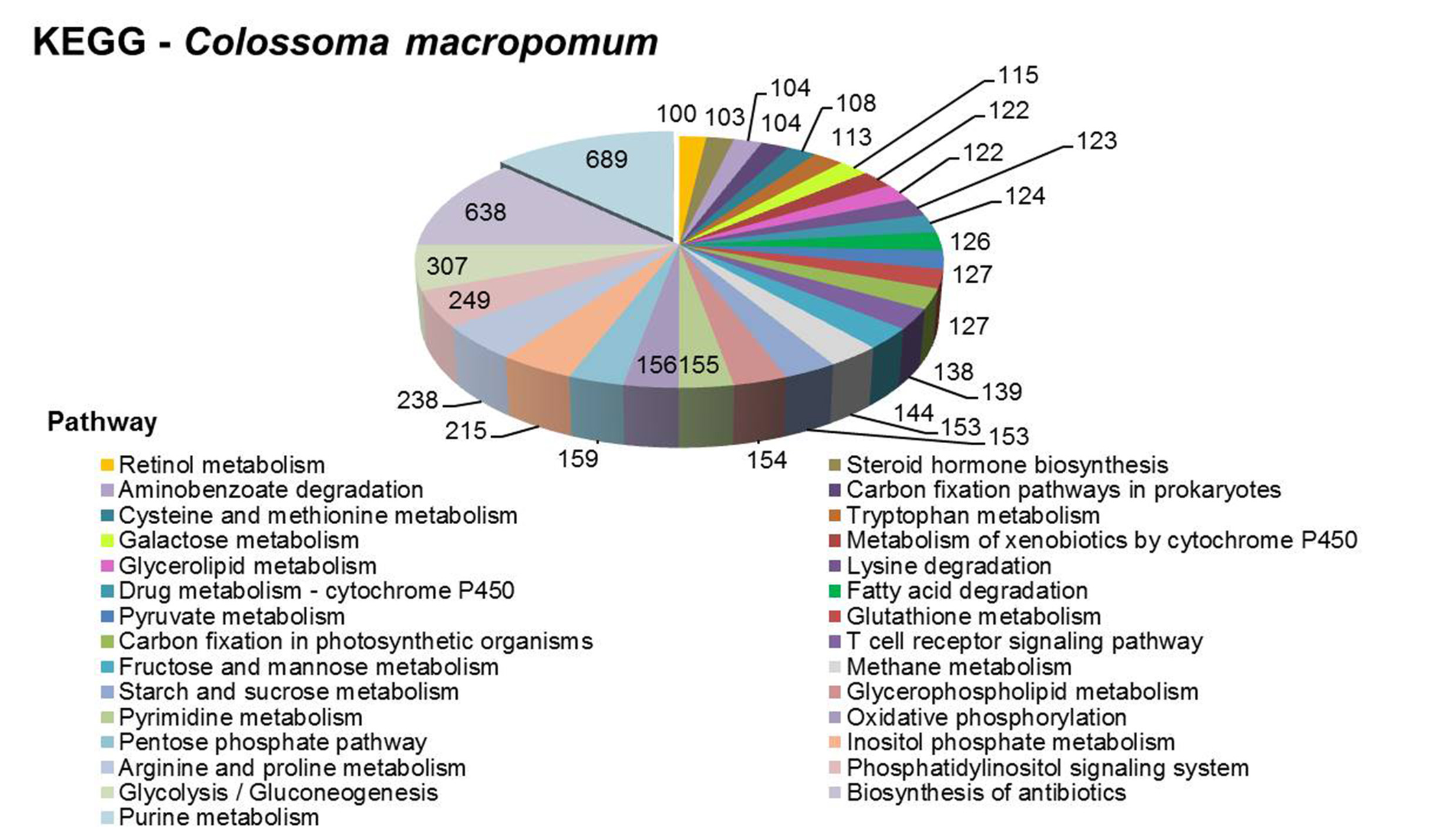

Supplement: S6 Fig — (TIF) [file pone.0212755.s006.tif]

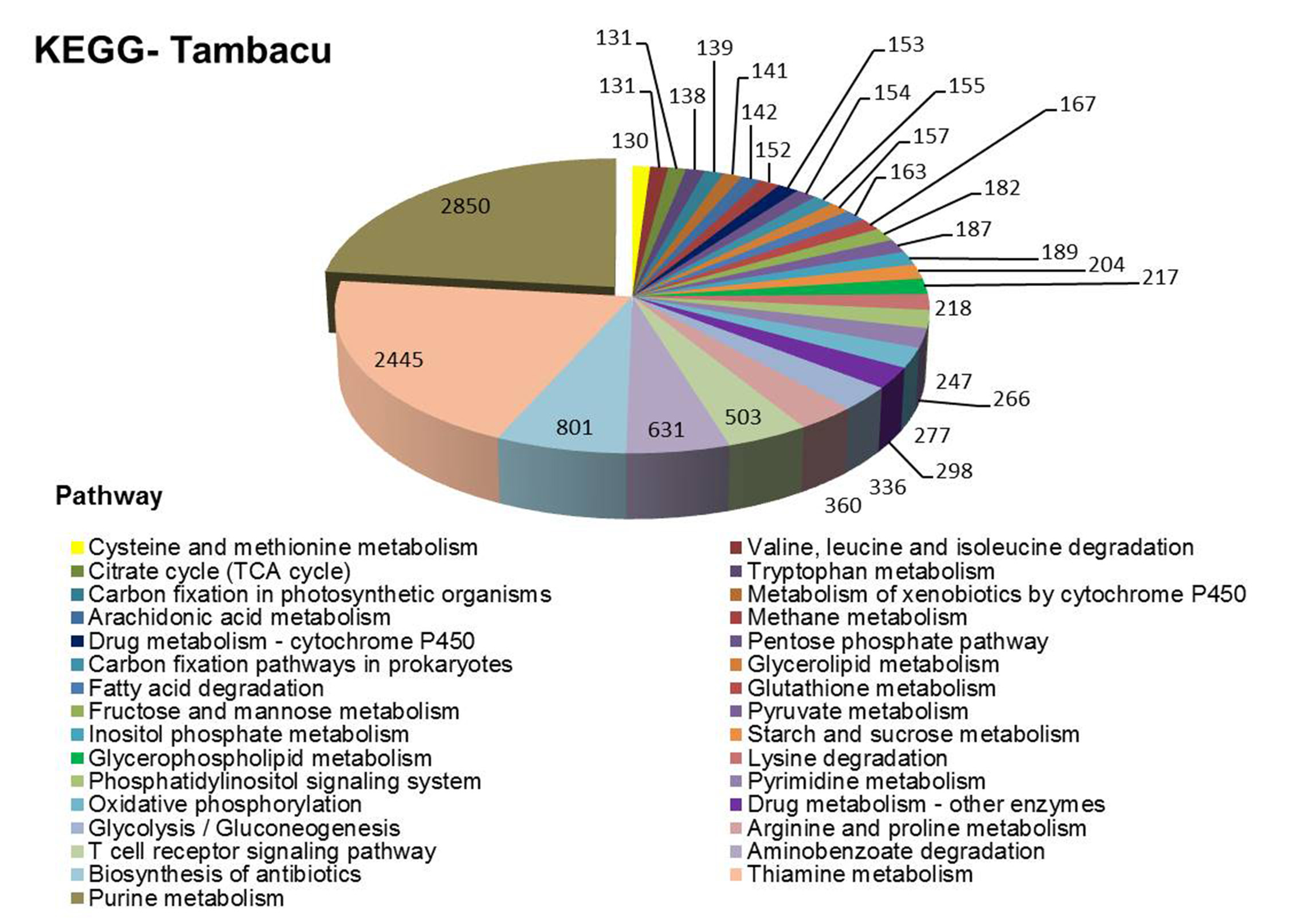

Supplement: S7 Fig — (TIF) [file pone.0212755.s007.tif]
